# Association of Follow-up Blood Cultures With Mortality in Patients With Gram-Negative Bloodstream Infections: A Systematic Review and Meta-analysis

**DOI:** 10.1001/jamanetworkopen.2022.32576

**Published:** 2022-09-22

**Authors:** Joshua T. Thaden, Sarah Cantrell, Michael Dagher, Yazhong Tao, Felicia Ruffin, Stacey A. Maskarinec, Stacy Goins, Matthew Sinclair, Joshua B. Parsons, Emily Eichenberger, Vance G. Fowler

**Affiliations:** 1 Duke University Division of Infectious Diseases, Durham, North Carolina; 2 Duke University Medical Center Library & Archives, Duke University School of Medicine, Durham, North Carolina; 3 Duke University School of Medicine, Durham, North Carolina; 4 Duke University Division of Nephrology, Durham, North Carolina

## Abstract

**Importance:**

Obtaining follow-up blood cultures (FUBCs) in patients with *Staphylococcus aureus* bloodstream infection (BSI) is standard practice, although its utility in patients with gram-negative bacterial BSI (GN-BSI) is unclear.

**Objective:**

To examine whether obtaining FUBCs is associated with decreased mortality (key question [KQ] 1) and whether positive vs negative FUBCs are associated with increased mortality (KQ2).

**Data Sources:**

MEDLINE, EMBASE, Cochrane Central Register of Controlled Trials, Web of Science, and gray literature were searched from inception to March 11, 2022.

**Study Selection:**

Two investigators used predefined eligibility criteria to independently screen titles, abstracts, and relevant full texts. Randomized clinical trials or observational studies that matched or statistically adjusted for differences in, at minimum, level of acute illness between patients in the intervention (eg, FUBCs obtained) and control (eg, FUBCs not obtained) groups were included in primary analyses. Articles published in languages other than English were excluded.

**Data Extraction and Synthesis:**

Data abstraction and quality assessments were performed by one investigator and verified by a second investigator. Risk of bias was assessed with the Newcastle-Ottawa Scale. Effect sizes were pooled using random-effects models. The study followed the Meta-analysis of Observational Studies in Epidemiology (MOOSE) reporting guideline.

**Main Outcomes and Measures:**

Mortality before hospital discharge or up to 30 days from the index blood culture.

**Results:**

From 3495 studies, 15 were included (all nonrandomized). In the 5 studies (n = 4378 patients) that met criteria for the KQ1 primary analysis, obtaining FUBCs was associated with decreased mortality (hazard ratio, 0.56; 95% CI, 0.45-0.71). For KQ2, 2 studies met criteria for the primary analysis (ie, matched or statistically adjusted for differences in patients with positive vs negative FUBCs), so an exploratory meta-analysis of all 9 studies that investigated KQ2 (n = 3243 patients) was performed. Positive FUBCs were associated with increased mortality relative to negative blood cultures (odds ratio, 2.27; 95% CI, 1.54-3.34). Limitations of the literature included a lack of randomized studies and few patient subgroup analyses.

**Conclusions and Relevance:**

In this systematic review and meta-analysis, obtaining FUBCs in patients with GN-BSI was associated with decreased mortality. The benefit of FUBCs may stem from identification of patients with positive FUBCs, which was a poor prognostic marker.

## Key Points

**Question:**

In patients with gram-negative bacterial bloodstream infections, is obtaining follow-up blood cultures after the initial positive blood cultures associated with decreased patient mortality?

**Findings:**

In this systematic review and meta-analysis that included 5 observational studies and 4378 patients in the primary analysis, obtaining follow-up blood cultures was associated with decreased patient mortality. The overall strength of evidence for the association of obtaining follow-up blood cultures with decreased mortality was moderate.

**Meaning:**

The findings of this study suggest that well-designed observational studies support the use of follow-up blood cultures in patients with gram-negative bloodstream infections; however, subgroup analyses that identify patients who do not require follow-up blood cultures are lacking.

## Teaser

This systematic review and meta-analysis examines the incidence of mortality in patients with gram-negative bacterial bloodstream infections who receive follow-up blood cultures.

## Introduction

Gram-negative bacterial bloodstream infection (GN-BSI) is a common medical problem associated with high mortality rates of 13% to 20%.^1,2^ Despite its frequency and severity, the optimal management of GN-BSI is unclear. For example, routinely obtaining follow-up blood cultures (FUBCs) to document clearance of bloodstream infection (BSI) is standard practice in patients with *Staphylococcus aureus* BSI but is controversial in patients with GN-BSI. Follow-up blood cultures can be helpful in determining whether control of the source of the infection has been achieved and antibiotic therapy is appropriate.^3,4,5^ However, FUBCs could also be associated with risks, including increased length of hospital stay and blood culture contaminants that unnecessarily complicate therapy.^6,7,8^ Data from published studies have not clearly established whether FUBCs performed to document clearance of BSI are necessary in patients with GN-BSI, because some,^5,7,9^ but not all,^6,10,11^ studies have reported an association between performance of FUBCs and decreased patient mortality. Thus, the role of FUBCs in the care for patients with GN-BSI is unknown.

This study is a systematic review and meta-analysis of randomized clinical trials and observational studies that investigated the association between obtaining FUBCs and mortality in patients with GN-BSI. This evidence synthesis may be used to inform the decision about whether to obtain such cultures.

## Methods

**Figure 1.**
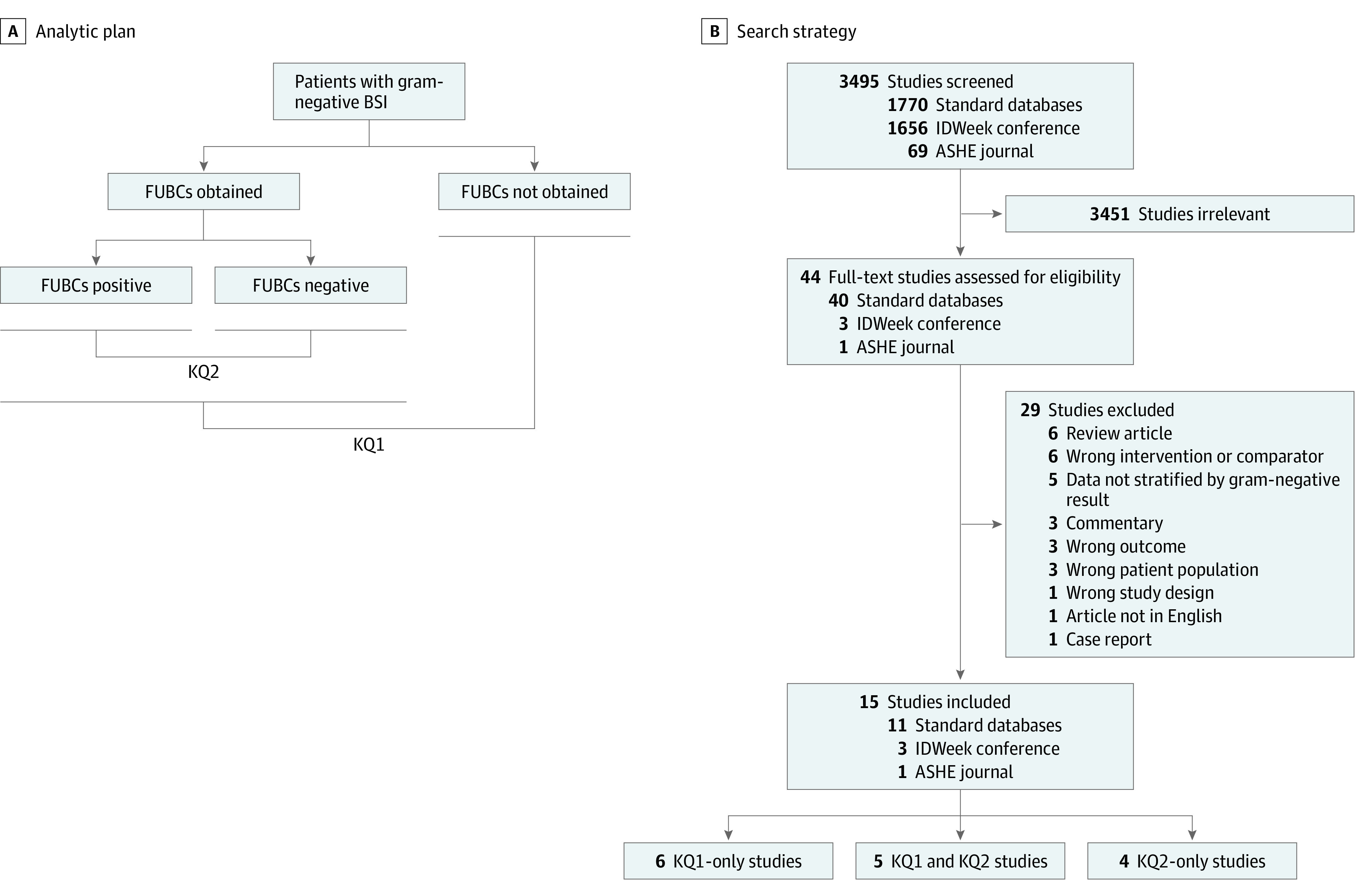
Analytic Plan and Search Strategy to Address Key Question (KQ) 1 and KQ2 ASHE indicates *Antimicrobial Stewardship and Healthcare Epidemiology*; BSI, bloodstream infection; and FUBCs, follow-up blood cultures.

This review focused on 2 key questions (KQs) (Figure 1A). Key question 1 was defined as follows: For patients with GN-BSI, is obtaining FUBCs after the initial positive blood cultures associated with decreased patient mortality? Key question 2 was defined as follows: For patients with GN-BSI and FUBCs, are positive FUBCs associated with increased mortality relative to negative FUBCs? We followed a standard protocol, developed before literature review, for all steps of this review (eAppendix 1 in the Supplement). We followed the Meta-analysis of Observational Studies in Epidemiology (MOOSE) reporting guideline.

### Search Strategy

We searched MEDLINE (via Ovid), the Cochrane Central Register of Controlled Trials (via Wiley), Embase (via Elsevier), and Web of Science–Science Citation Index and Social Sciences Citation Index (via Clarivate) from inception to February 11, 2022, using a combination of database-specific subject headings and key words searched in the title and abstract for the following concepts: GN-BSI, follow-up, and blood cultures. One of us, an experienced medical librarian (S.C.), devised and conducted the search, with input from the rest of us and from another librarian, using the Peer Review of Electronic Search Strategies Checklist.^12^ No limitations were placed on language in the initial search, but studies published in languages other than English were excluded in the full-text review phase. A search update was conducted on March 11, 2022, to identify newly published studies. The search strategies for all included databases are given in eAppendix 2 in the Supplement. We manually searched the journal *Antimicrobial Stewardship and Healthcare Epidemiology*, because it was not indexed in the above databases at the time of the search. To be fully comprehensive and incorporate emerging studies not yet published, we manually reviewed gray literature, including titles and abstracts from the IDWeek 2021 conference.^13^ Authors were contacted to obtain missing data. We hand-searched key references to identify citations not captured in the electronic database searches.^14,15,16,17,18^ We searched ClinicalTrials.gov to identify completed but unpublished studies meeting our eligibility criteria to reduce publication bias. All citations were imported into Covidence, a systematic review screening software.^19^

### Study Selection, Data Extraction, and Quality Assessment

Two reviewers (J.T.T. reviewed all; M.D., Y.T., F.R., S.A.M., S.G., M.S., J.B.P., and E.E. were second reviewers) used prespecified eligibility criteria to independently screen titles and abstracts (eAppendix 3 in the Supplement). Eligibility criteria included studies evaluating adult inpatients with GN-BSI that stratified mortality by either whether FUBCs were obtained (KQ1) or whether FUBCs were positive (ie, growth of same bacterial species in FUBC and index blood culture) or negative (KQ2). Included articles were independently screened by 2 reviewers at the full-text level. Disagreements on inclusion at both stages of screening were resolved by discussion. Studies excluded in the full-text review are listed in eAppendix 4 in the Supplement. Data abstraction and quality assessment were performed by one investigator (J.T.T.) and verified by a second investigator (M.D., Y.T., and S.G.). Risk of bias and quality were assessed with the Newcastle-Ottawa Quality Assessment Scale,^20^ because only observational studies were identified (eAppendix 5 in the Supplement).

### Data Synthesis and Analysis

For both KQ1 and KQ2, the primary analysis focused on randomized clinical trials or observational studies that either matched or statistically adjusted for differences in, at a minimum, the level of acute illness between patients in the intervention (eg, FUBCs obtained) and control (eg, FUBCs not obtained) groups. Such matching or statistical adjustment in nonrandomized studies is necessary, because multiple studies have reported that patients with and without FUBCs differ with respect to level of acute illness, age, source of BSI, and other factors that influence mortality.^5,6,7,9,11,21^

When quantitative analysis (ie, meta-analysis) of the adjusted estimate effects (eg, hazard ratios [HRs]) were feasible (ie, ≥3 studies in analysis), estimates were combined using inverse variance with random-effects models. One study reported a mortality odds ratio (OR),^22^ and this was converted to a mean HR across the time period of interest. Dichotomous data (eg, unadjusted mortality) were combined as ORs using Mantel-Haenszel analysis with random-effects models. We used the Knapp and Hartung method to adjust the SEs of the estimated coefficients.^23,24^ One study contained censored data,^9^ and censored patients were removed so that only patients with complete follow-up information were included. Robustness of the findings was assessed through influence and sensitivity analyses as detailed in the Results section. We evaluated statistical heterogeneity with the Cochran *Q* and *I*^2^ statistics. When quantitative synthesis was not feasible, we analyzed data qualitatively. Statistical analyses were performed with RStudio 2022.02.0.

Publication bias was assessed using funnel plots with the Egger test^25^ when 10 or more studies were included in the analysis. We used the Evidence-based Practice Center model from the US Agency for Healthcare Research and Quality to grade overall strength of evidence.^26^ A full description of the Evidence-based Practice Center approach is detailed in the eMethods in the Supplement. Findings at *P* < .05 in unpaired, 2-sided testing were considered significant.

## Results

### Summary of Included Studies

We screened 3495 studies, and 3451 were deemed irrelevant at the title and abstract screening phase. A total of 44 studies were assessed at the full-text level, and 29 were excluded. We identified 15 studies for inclusion (Figure 1B). Six studies addressed only KQ1,^6,7,10,21,22,27^ 4 addressed only KQ2,^28,29,30,31^ and 5 studies addressed both KQs.^5,8,9,11,32^ Study and patient characteristics of included studies are reported in the Table. There were 8007 patients in studies that addressed KQ1 and 3243 patients in studies that addressed KQ2. No randomized clinical trials were identified. The nonrandomized studies were mainly cohort studies (KQ1: 11 of 11 [100%]; KQ2: 8 of 9 [89%]). There was substantial variability in how FUBCs were defined. The most common definition of FUBCs were those drawn between 24 hours and 7 days after index culture (KQ1: 6 of 11 [55%]; KQ2: 3 of 9 [33%]). A detailed description of studies that addressed KQ1 and KQ2 is provided in eTable 1 in the Supplement. All 5 studies that met criteria for our KQ1 primary analysis addressed survival bias through 1 or more of the following approaches: exclusion of patients who died within 72 hours of the index positive blood culture,^9,22^ treated FUBCs as a time-dependent covariate,^5,7,21^ and matching patients with and without FUBCs such that patients who did not have FUBCs survived at least as long as it took for the matched patient to have FUBCs^7^ (eTable 1 in the Supplement). Five studies that addressed KQ1 (5 of 11 [45%]) and 4 studies that addressed KQ2 (4 of 9 [44%]) were rated as having low risk of bias. A detailed quality assessment of each included study is given in eAppendix 6 in the Supplement. A funnel plot to assess publication bias was performed for all studies that addressed KQ1 and did not reveal significant bias (eFigure 1 in the Supplement).

**Table.** Study and Patient Characteristics of Included Studies

Characteristic	No. (%)	
	KQ1 (n = 11)	KQ2 (n = 9)
Patients	8007	3243
Design		
Cohort study	11 (100)	8 (89)
Case-control study	0	1 (11)
Study setting		
All inpatients	9 (82)	8 (89)
Inpatients with community-acquired BSI	1 (9)	0
Oncology units	1 (9)	0
ICU	0	1 (11)
Study population		
All patients with GN-BSI	9 (82)	8 (89)
Immunocompromised	2 (18)	1 (11)
Microbiology		
All GN-BSI	9 (82)	7 (78)
Only *Pseudomonas aeruginosa* BSI	1 (9)	0
Only *E coli* or Klebsiella BSI	1 (9)	2 (22)
Country, No. of studies		
US	8 (73)	5 (56)
Canada	1 (9)	1 (11)
South Korea	1 (9)	2 (22)
Italy	1 (9)	1 (11)
Country, No. of patients		
US	4049 (51)	2054 (63)
Canada	901 (11)	247 (8)
South Korea	1481 (18)	893 (28)
Italy	1576 (20)	49 (2)
No. of hospitals in study		
1	7 (64)	5 (56)
2	2 (18)	3 (33)
3	1 (9)	0
4	1 (9)	1 (11)
Definition of FUBC		
Drawn between 24 h and 7 d after index culture	6 (55)	3 (33)
Drawn between 24 h and 4 d after index culture	1 (9)	1 (11)
Drawn between 4 h and 3 d after index culture	1 (9)	0
Drawn 2-7 d after index culture	2 (18)	1 (11)
Drawn 2-3 d after index culture	0	1 (11)
Drawn >24 h after index culture	0	1 (11)
Drawn <48 h after start of antibiotics	0	1 (11)
Not reported	1 (9)	1 (11)
Outcome measure^a^		
In-hospital mortality	6 (55)	4 (44)
28-d mortality	1 (9)	1 (11)
30-d mortality	5 (45)	4 (44)
Risk of bias/quality		
Low/good	5 (45)	4 (44)
Medium/fair	0	0
High/poor	6 (55)	5 (56)

Abbreviations: BSI, bloodstream infections; FUBC, follow-up blood culture; GN, gram-negative; ICU, intensive care unit; KQ, key question.

^a^ One study reported more than 1 outcome measure.

### KQ1 Outcomes

**Figure 2.**
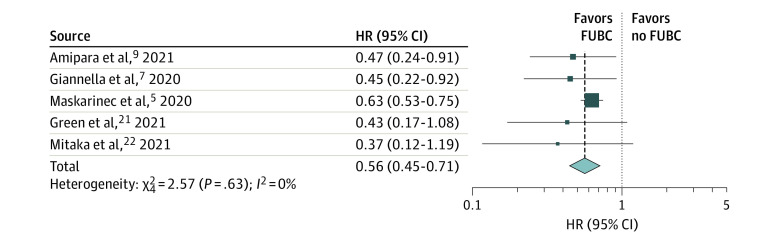
Association of Obtaining Follow-up Blood Cultures (FUBCs) With Mortality in Patients With Gram-Negative Bloodstream Infection HR indicates hazard ratio.

Five studies (n = 4378 patients) met the criteria for our primary KQ1 analysis. Obtaining FUBCs was associated with decreased mortality in patients with GN-BSI (HR, 0.56; 95% CI, 0.45-0.71) (Figure 2). Little heterogeneity was observed in this analysis (*Q* = 2.3; *P* = .68; *I*^2^ = 0%). Stratification by source of the study (ie, peer-reviewed journal vs conference abstract) revealed no major change in study findings (eFigure 2 in the Supplement). All 5 studies in this analysis were rated as low risk of bias and good quality, and thus no stratification by study quality was necessary. All 5 studies in this analysis addressed survival bias and so no subgroup analysis was necessary. An influence analysis yielded no major changes in findings with omission of any single study (eFigure 2 in the Supplement). Only the study of Amipara et al^9^ contained censored data, and so stratification by studies with complete follow-up data was contained within the influence analysis (ie, omitting Amipara et al^9^ in eFigure 2 in the Supplement).

As an exploratory analysis, we performed a meta-analysis with the unadjusted mortality data from the 11 studies that addressed KQ1. There was substantial heterogeneity in this analysis (*Q* = 24.6; *P* = .006; *I*^2^ = 59%) (eFigure 3 in the Supplement). Obtaining FUBCs was not associated with decreased mortality (OR, 0.75; 95% CI, 0.54-1.04). We hypothesized that the heterogeneity in this meta-analysis stemmed in part from institutional variation in FUBC practices. For example, institutions that typically obtain FUBCs for only the sickest patients will have a low percentage of patients with FUBCs and relatively higher unadjusted mortality in patients with FUBCs. Giannella et al^7^ stood out in this regard as it had by far the lowest percentage of patients with FUBCs (18% vs 64% collectively in all other studies). Furthermore, unadjusted mortality was higher in patients with FUBCs in the Giannella et al^7^ study (14% vs 11%), despite multiple analyses that adjusted for patient comorbidities and acute illness showing that obtaining FUBCs was associated with decreased mortality in this cohort (logistic regression: HR, 0.48; 95% CI, 0.27-0.83; *P* = .001; matching by level of acute illness [Sequential Organ Failure Assessment Score]: HR, 0.45; 95% CI, 0.22-0.92; *P* = .03). This study illustrates the challenge of using unadjusted mortality in observational studies to assess mortality risk in patients with and without FUBCs. Omission of Giannella et al^7^ resulted in lower heterogeneity (*Q* = 13.5; *P* = .14; *I*^2^ = 33%) and an association between obtaining FUBCs and decreased mortality (OR, 0.68; 95% CI, 0.52-0.90) (eFigure 3 in the Supplement). An influence analysis showed that Giannella et al^7^ was the study most responsible for increasing heterogeneity (eFigure 3 in the Supplement).

### KQ2 Outcomes

Nine studies addressed KQ2. Only 2 studies met the criteria for the primary analysis (ie, adjusted for level of acute illness between patients who had positive vs negative FUBCs) and so no meta-analysis was performed. Both studies showed an association between positive FUBCs and increased mortality in patients with GN-BSI. Kim et al^30^ performed a multivariable logistic regression analysis of 30-day mortality in patients who had positive FUBCs (n = 89) vs negative FUBCs (n = 556) and found that positive FUBCs were associated with increased mortality (OR, 2.01; 95% CI, 1.07-3.76; *P* = .03). Maskarinec et al^5^ performed an inverse probability of weighting approach for matching patients with positive (n = 228) and negative (n = 885) FUBCs. A Cox proportional hazards regression analysis on this weighted cohort similarly showed that positive FUBCs were associated with increased mortality relative to negative FUBCs (HR, 1.56; 95% CI, 1.13-2.16; *P* = .007).

**Figure 3.**
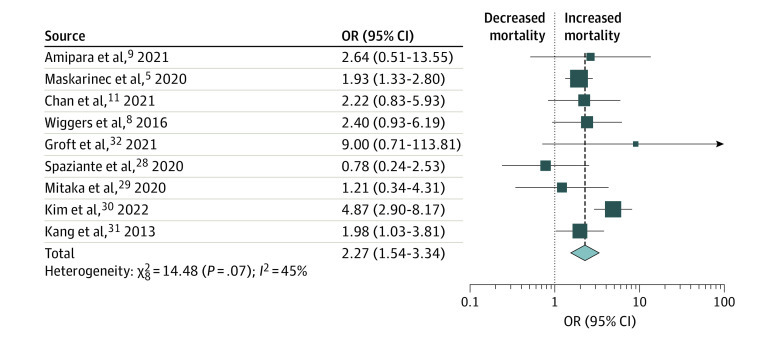
Association of Positive Follow-up Blood Cultures (FUBCs) With Mortality in Patients With Gram-Negative Bloodstream Infection The mortality odds ratio (OR) associated with positive FUBCs, relative to negative FUBCs, is presented.

As an exploratory analysis, we performed a meta-analysis with the unadjusted mortality data from the 9 studies that addressed KQ2. Positive FUBCs were associated with increased mortality compared with negative FUBCs (OR, 2.27; 95% CI, 1.54-3.34). Moderate heterogeneity was present (*Q* = 14.5; *P* = .07; *I*^2^ = 45%) (Figure 3). An influence analysis and stratification by study type or study quality yielded no major changes in results (eFigure 4 in the Supplement). Omission of Kim et al^30^ alone was sufficient to substantially decrease the heterogeneity of the meta-analysis (*Q* = 4.6; *P* = .71; *I*^2^ = 0%). Kim et al^30^ did not differ substantially from other studies in terms of design or patient population, although it did identify particularly high mortality among patients with positive vs negative FUBCs (35% vs 10%).

### Subgroup Analysis

There was little investigation into the association between mortality and either obtaining FUBCs (KQ1) or positive vs negative FUBCs (KQ2) among patient subgroups. Green et al,^21^ a cohort study of 159 patients with *Pseudomonas aeruginosa* BSI, addressed KQ1 in patients with varying levels of acute illness. In the overall cohort, this study showed that FUBCs were associated with a nonsignificant reduction in mortality risk after adjustment for patient characteristics (HR, 0.43; 95% CI, 0.17-1.08; *P* = .07). Kaplan-Meier survival curves were generated for patients with low (score, 0), medium (scores, 1-4), and high (scores, ≥5) Pitt bacteremia scores. Survival curves were compared by log-rank test without adjustment for confounding variables. Follow-up blood cultures were associated with a significant decrease in mortality among patients with medium Pitt bacteremia scores and a nonsignificant decrease in mortality among those with high Pitt bacteremia scores. In patients with low Pitt bacteremia scores, there was no discernable association between FUBCs and mortality. No studies investigated KQ2 among subgroups of patients with GN-BSI.

### Evaluation of the Evidence

The evidence profile shows the overall strength of evidence from the KQ1 primary analysis (eTable 2 in the Supplement). Given that this systematic review contained well-designed observational studies that accounted for confounding through matching or statistical adjustment, the baseline strength of evidence was moderate. The KQ1 mortality effect estimate was not downrated due to risk of bias, inconsistency, indirectness, imprecision, or publication bias. We did not have serious concerns about risk of bias because all studies had low risk of bias according to the Newcastle-Ottawa Quality Assessment Scale. We also did not have serious concerns for inconsistency or imprecision, because all 5 studies had similar HRs and the 95% CI of the pooled estimate was narrow. We did not have concern for indirectness, because the evidence directly answered the question that was asked. We did not detect publication bias. Therefore, the overall strength of evidence for the association of obtaining FUBCs with decreased mortality in patients with GN-BSI was moderate.

## Discussion

In this systematic review and meta-analysis, we addressed the role of FUBCs in managing treatment in patients with GN-BSI, which is an area of controversy. This study revealed that obtaining FUBCs was associated with decreased mortality, highlighted the challenges of using unadjusted mortality data in patients from observational studies, and noted that limited subgroup analyses have been performed to identify patients who do not require FUBCs. These findings may have major implications for routine management of GN-BSI, a serious, common infection.

First, obtaining FUBCs was associated with decreased mortality in patients with GN-BSI. The included studies had a consistent association between obtaining FUBCs and decreased mortality with little heterogeneity. This study did not directly address the mechanism by which obtaining FUBCs could influence patient mortality. However, given the complementary finding that persistent bloodstream infection (ie, positive FUBCs) was associated with increased mortality relative to nonpersistent bloodstream infection (ie, negative FUBCs), it is tempting to speculate that recognition of patients with positive FUBCs could identify those with complicated GN-BSI and correctable issues in GN-BSI management, such as a lack of source control or inappropriate antibiotic therapy. This area requires further study.

Second, this study highlights the challenges in interpreting unadjusted mortality data in patients from observational studies. We focused on studies that performed matching or statistical adjustment between the intervention (eg, FUBCs) and control (eg, no FUBCs) populations because there is clear selection bias between the 2 groups. The problem of selection bias is illustrated most by Giannella et al,^7^ in which FUBCs were obtained in a relatively small percentage of sick patients. Although the unadjusted mortality data would suggest that patients with FUBCs have increased mortality, multiple analyses that adjusted for patient level of acute illness and other covariates clearly showed an association between obtaining FUBCs and decreased mortality. It may be useful for future studies to perform matching or statistical adjustment to reduce confounding by important variables, such as level of acute illness, age, and source of BSI.

Third, this study did not identify groups of patients with GN-BSI that do not require FUBCs, although we acknowledge that such subpopulations likely exist. Only Green et al^21^ examined the association of obtaining FUBCs with mortality among patient subgroups with varying levels of acute illness. Green et al^21^ made the intriguing finding that the mortality benefit of obtaining FUBCs was only notable for patients with a Pitt bacteremia score greater than 0. Although this finding requires validation, it is a simple and potentially useful way to stratify patients who do or do not require FUBCs. However, detecting statistically significant differences among patients with a Pitt bacteremia score of 0 would be challenging given the low estimated mortality in this group and the high number of patients who would need to be enrolled in an adequately powered study.

### Limitations

This study has limitations. We identified no randomized clinical trials and so must rely on observational data. The primary analyses focused on observational data that accounted for selection and survival bias through matching, statistical adjustment, and exclusion criteria, although we cannot fully assess how well these approaches minimized such biases. Multiple observational studies did not perform matching or statistical adjustment for patients in the intervention and control arms, and inclusion of matched and adjusted data from these cohorts could have influenced study results. No meta-analysis of individual patient-level data was attempted because the included studies differed substantially in the covariates used to describe chronic medical comorbidities and level of acute illness. There was a general lack of studies that addressed the association of FUBCs with mortality in patient subgroups. Such studies are critical to better understand which patients with GN-BSI do and do not require FUBCs. Only English-language studies were included, and this may have biased the results.

## Conclusions

In this systematic review and meta-analysis, well-designed observational cohort studies showed an association between FUBCs and mortality in patients with GN-BSI. Although there are likely patient populations for which FUBCs are not associated with mortality, to our knowledge, studies to date have not defined these subgroups. It is tempting to suggest that randomized clinical trials are needed to further address this issue given that residual confounding could account for the observed results, although we question whether equipoise is present between study arms given the findings of this systematic review. Furthermore, for such a trial to be of benefit, it would require a large number of sick patients (ie, elevated Pitt bacteremia scores) with GN-BSI, a population that has traditionally been difficult to successfully enroll into trials.

## Notes

Supplement. **eAppendix 1.** Systematic Review Protocol **eAppendix 2.** Search Strategies **eAppendix 3.** Criteria for Inclusion and Exclusion of Studies in This Systematic Review **eAppendix 4.** Studies Excluded at Full-Text Review **eAppendix 5.** Newcastle-Ottawa Quality Assessment Scale for Assessing Risk of Bias in Cohort (A) and Case-Control (B) Studies **eTable 1.** Detailed Description of Studies That Address KQ1 and KQ2 **eAppendix 6.** Risk of Bias (Quality) Assessment of Included Cohort (A) and Case-Control (B) Studies Using the Newcastle-Ottawa Quality Assessment Scale **eFigure 1.** Funnel Plot for Key Question 1 **eFigure 2.** Robustness of KQ1 Primary Analysis **eFigure 3.** Exploratory Analysis of KQ1 With Unadjusted Mortality Data **eFigure 4.** Robustness of KQ2 Exploratory Analysis **eTable 2.** Evidence Profile for Association of Obtaining Follow-up Blood Cultures With Mortality in Patients With Gram-Negative Bloodstream Infections **eMethods.** Approach for Grading the Overall Strength of Evidence
eReferences
