# Supplementary material for: Association of Follow-up Blood Cultures With Mortality in Patients With Gram-Negative Bloodstream Infections: A Systematic Review and Meta-analysis
Source: JAMA Netw Open. 2022 Sep 22;5(9):e2232576. doi: 10.1001/jamanetworkopen.2022.32576 (PMC9500561; doi:10.1001/jamanetworkopen.2022.32576)
Supplement: Supplement. — eAppendix 1. Systematic Review Protocol eAppendix 2. Search Strategies eAppendix 3. Criteria for Inclusion and Exclusion of Studies in This Systematic Review eAppendix 4. Studies Excluded at Full-Text Review eAppendix 5. Newcastle-Ottawa Quality Assessment Scale for Assessing Risk of Bias in Cohort (A) and Case-Control (B) Studies eTable 1. Detailed Description of Studies That Address KQ1 and KQ2 eAppendix 6. Risk of Bias (Quality) Assessment of Included Cohort (A) and Case-Control (B) Studies Using the Newcastle-Ottawa Quality Assessment Scale eFigure 1. Funnel Plot for Key Question 1 eFigure 2. Robustness of KQ1 Primary Analysis eFigure 3. Exploratory Analysis of KQ1 With Unadjusted Mortality Data eFigure 4. Robustness of KQ2 Exploratory Analysis eTable 2. Evidence Profile for Association of Obtaining Follow-up Blood Cultures With Mortality in Patients With Gram-Negative Bloodstream Infections eMethods. Approach for Grading the Overall Strength of Evidence eReferences [file jamanetwopen-e2232576-s001.pdf]

Supplementary Online Content

Thaden JT, Cantrell S, Dagher M, et al. Association of follow-up blood cultures with mortality in patients with gram-negative bloodstream infections: a systematic review and meta-analysis. *JAMA Netw Open*. 2022;5(9):e2232576. doi:10.1001/jamanetworkopen.2022.32576

eAppendix 1. Systematic Review Protocol

eAppendix 2. Search Strategies

eAppendix 3. Criteria for Inclusion and Exclusion of Studies in This Systematic Review

eAppendix 4. Studies Excluded at Full-Text Review

eAppendix 5. Newcastle-Ottawa Quality Assessment Scale for Assessing Risk of Bias in Cohort (A) and Case-Control (B) Studies

eTable 1. Detailed Description of Studies That Address KQ1 and KQ2

eAppendix 6. Risk of Bias (Quality) Assessment of Included Cohort (A) and Case-Control (B) Studies Using the Newcastle-Ottawa Quality Assessment Scale

eFigure 1. Funnel Plot for Key Question 1

eFigure 2. Robustness of KQ1 Primary Analysis

eFigure 3. Exploratory Analysis of KQ1 With Unadjusted Mortality Data

eFigure 4. Robustness of KQ2 Exploratory Analysis

eTable 2. Evidence Profile for Association of Obtaining Follow-up Blood Cultures With Mortality in Patients With Gram-Negative Bloodstream Infections

eMethods. Approach for Grading the Overall Strength of Evidence

eReferences

This supplementary material has been provided by the authors to give readers additional information about their work.

© 2022 Thaden JT et al. *JAMA Network Open.*

eAppendix 1. Systematic review protocol.

Project Title: Association of follow-up blood cultures with mortality in patients with gram-negative bloodstream infections

Investigators: Joshua T. Thaden, MD, PhD; Felicia Ruffin, PhD; Stacey A. Maskarinec, MD, PhD; Stacy Goins, BA; Matthew Sinclair, MD; Joshua Parsons, MD, PhD; Yazhong Tao, PhD; Emily Eichenberger, MD, Michael Dagher, MD; Vance G. Fowler, Jr., MD, MHS
Search Librarian: Sarah Cantrell, MLIS, AHIP

I. Background

Bloodstream infection (BSI) with gram-negative bacteria is a common medical problem that is associated with high morbidity and mortality. However, there are ongoing controversies on how patients with gram-negative BSI should be best managed. For example, data from published studies has not clearly established whether follow-up blood cultures to document clearance of BSI are necessary in patients with gram-negative BSI. Some studies have shown that getting follow-up blood cultures to document clearance of the BSI is associated with decreased mortality,[1-3] while other studies have not shown an association between follow-up blood cultures and mortality.[4-6]

The purpose of this project is to conduct a systematic review of randomized controlled trials and observational studies assessing the association between obtaining follow-up blood cultures and mortality in patients with gram-negative BSI. This evidence synthesis will be used to inform the decision about whether to obtain such cultures.

<u>Project Timeline</u>
Kickoff call: February 1, 2022
Project Start: February 14, 2022
Anticipated draft manuscript due: April 1, 2022
Anticipated manuscript submission: May 1, 2022

II. Key question

The key research questions for this systematic review were developed after a topic refinement process that included a preliminary review of published, peer-reviewed literature; consultation with internal partners and investigators; and consultation with content experts.

The key questions (KQ) for this systematic review are:

KQ 1: For patients with gram-negative bloodstream infections, is obtaining follow-up blood cultures after the initial positive blood culture(s) associated with decreased patient mortality?

KQ 2: For patients with gram-negative bloodstream infection and follow-up blood cultures, are positive follow-up blood cultures associated with increased mortality relative to negative follow-up blood cultures?

A. Population

The population will be hospitalized adults (age 18 years and older) with gram-negative BSI. Studies that include patients with both gram-negative and gram-positive BSI will be included so long as the gram-negative cohort is analyzed separately.

B. Intervention

KQ1: The intervention is obtaining a follow-up blood culture after the index blood culture that is positive with the gram-negative bacteria. We will not specify the time range in which the follow-up blood culture must be drawn, though in general studies define follow-up blood cultures as those drawn 1-7 days after the index positive culture.

KQ2: Not necessarily an intervention per se, but the experimental group is patients with gram-negative BSI who had a positive follow-up blood culture.

© 2022 Thaden JT et al. *JAMA Network Open.*

C. Comparators

KQ1: The comparator group will be patients with gram-negative BSI and no follow-up blood cultures.

KQ2: The comparator group will be patients with gram-negative BSI who had negative follow-up blood cultures.

D. Outcomes

The outcome will be mortality.

E. Timing

In-hospital mortality or mortality up to 30 days.

F. Settings

Patients will be admitted to the hospital.

III. Analytical Framework

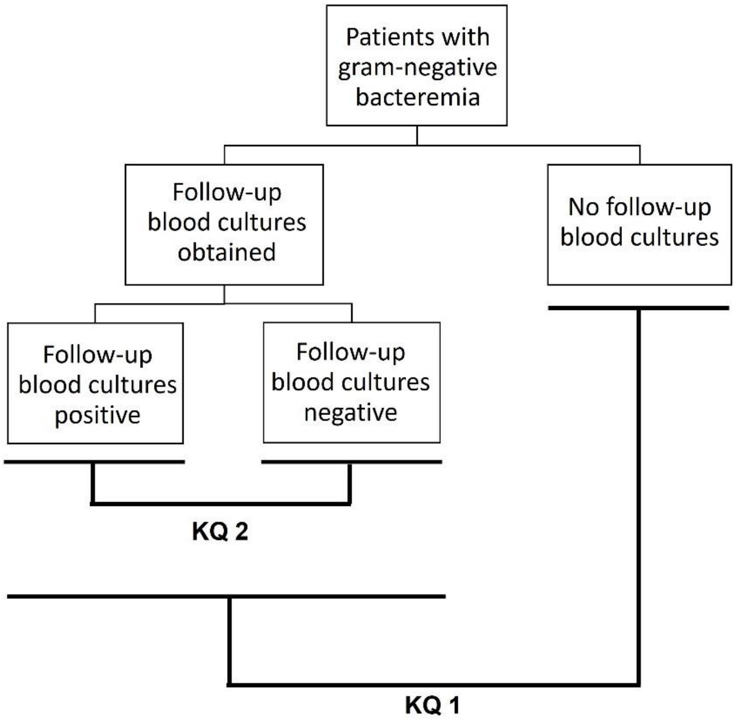

IV. Methods

A. Criteria for inclusion and exclusion of studies in this systematic review

Inclusion criteria

- Study design: Randomized controlled trials or observational studies with N>10. The sample size requirement is designed to exclude case reports. This size requirement does permit smaller pilot studies that are typically underpowered and have more methodological problems than larger studies. In order to account for differences in methodology between the studies, we will perform analyses with two sets of studies: 1) the entire set of papers identified through this systematic review, and 2) the subset of studies identified

© 2022 Thaden JT et al. *JAMA Network Open.*

through this systematic review that meet a baseline level of methodological rigor. The methodological requirements for inclusion in this second group are defined below in section IV.E.

- Age: Adults aged 18 years or older with gram-negative BSI
- Setting: Inpatients
- Studies must stratify patients by whether follow-up blood cultures obtained (KQ1) or by whether follow-up blood cultures were positive or negative (KQ2)
- Study must report effects of follow-up blood cultures on at least one of the following relevant outcomes:
    - In-hospital mortality
    - Mortality up to 30 days (e.g., 28-day mortality, 30-day mortality)
- Article is a full publication, research letter, or meeting abstract published in a peer-reviewed journal.
- Article is an English language publication

Exclusion criteria

- Not an English language publication
- Outpatient setting
- Polymicrobial BSI

B. Literature search strategies

We will conduct a primary search of MEDLINE (via Ovid), the Cochrane Central Register of Controlled Trials (via Wiley), Embase (via Elsevier), and Web of Science – Science Citation Index and Social Sciences Citation Index (via Clarivate). We will further evaluate the bibliographies of any systematic or nonsystematic reviews for relevant studies. We will manually review titles and abstracts from the journal *Antimicrobial Stewardship and Healthcare Epidemiology* as it is a new journal that is not indexed in the above databases at the time of this search. In an effort to be fully comprehensive, we will manually review abstracts from the most recent IDWeek conference to identify emerging research that has not yet been published. We anticipate using a combination of database-specific controlled vocabulary and selected free-text terms to search titles and abstracts. To ensure completeness, search strategies will be developed and executed by a medical librarian, with input on terms from the research team. We will also search ClinicalTrials.gov to identify completed but unpublished studies meeting our eligibility criteria in an effort to reduce publication bias.

Using pre-specified inclusion/exclusion criteria, titles and abstracts of articles included in the existing reviews and identified through our primary search will be reviewed independently by two reviewers for potential relevance to the KQs. Articles included by any two reviewers will undergo full-text screening. Conflicts at this stage will be resolved by a third person. At the full-text screening stage, articles will be reviewed independently by two reviewers. The screeners must agree on a final inclusion/exclusion decision. Conflicts at this stage will be resolved by a third person. Articles meeting eligibility criteria will be included for data abstraction. All results will be tracked in Covidence, a web-based data synthesis software program, and, for manuscript preparation, EndNote X20 reference management software (Thomson Reuters).

C. Data abstraction and data management

Data from published reports will be abstracted into a customized Covidence data extraction form by one reviewer and overread by a second reviewer. Disagreements will be resolved by consensus or by obtaining a third reviewer's opinion when consensus cannot be reached. Data elements include descriptors to assess applicability, quality elements, intervention/exposure details, and outcomes.

Key characteristics abstracted will include patient descriptors (including age, gender, race, level of acute illness, source of BSI), setting, definitions of the intervention, and outcomes as described previously. We will capture any subgroup analyses of particular interest, including differential intervention effects by level of acute illness. When critical data are missing or are unclear in published reports, we plan to request supplemental data from the study authors. Key features relevant to applicability include the match between the sample and target populations and statistical adjustment for patient comorbidities.

© 2022 Thaden JT et al. *JAMA Network Open.*

D. Assessment of methodological quality of individual studies

Quality assessment will be performed by the researcher abstracting or evaluating the included article; this initial assessment will then be overread by a second, highly experienced reviewer. Disagreements will be resolved between the two reviewers or when needed by arbitration from a third reviewer.

We will use the key quality criteria described in the Agency for Healthcare Research and Quality's (AHRQ's) "Methods Guide for Effectiveness and Comparative Effectiveness Reviews" adapted to this specific topic. We will assign a summary quality score (good, fair, or poor) to individual studies.

E. Data synthesis

We will summarize the primary literature by abstracting the relevant data. We will develop a summary table describing the key outcomes and the types of study designs used to evaluate the association of follow-up blood cultures and mortality in patients with gram-negative BSI (KQ1) and the association between positive follow-up blood cultures and mortality in patients with gram-negative BSI (KQ2). We will then determine the feasibility of completing a quantitative synthesis (i.e., meta-analysis) to estimate summary effects for each KQ. Feasibility depends on the volume of relevant literature, conceptual homogeneity of the studies, and completeness of results reporting. We will aggregate outcomes when there are at least three studies with the same outcome, based on the rationale that one or two studies do not provide adequate evidence for summary effects. If meta-analyses are feasible, we will explore the possibility of subgroup analyses to explore the consistency of effects across conditions (e.g., different levels of acute illness). If such subgroup analyses are possible, we will stratify analyses to determine how follow-up blood cultures impact mortality in the individual subgroups (e.g., high level of acute illness versus lower level of acute illness). We recognize that subgroup analyses involve indirect comparisons (across studies) and are subject to confounding.

Studies included in this systematic review will report the dichotomous outcome of mortality. If quantitative synthesis is possible, mortality will be reported using risk ratio or odds ratio. We will evaluate for statistical heterogeneity using visual inspection and Cochrane's Q and I^2 statistics. Publication bias will be assessed using findings from the ClinicalTrials.gov search (described previously) and using funnel plots (when there are >10 studies in an analysis).

If a quantitative synthesis is not feasible, we will analyze the data qualitatively. We will give more weight to the evidence from higher quality studies with more precise estimates of effect. A qualitative synthesis would focus on documenting and identifying patterns in efficacy and safety of the intervention. We will analyze potential reasons for inconsistency in treatment effects across studies by evaluating differences in the study population, intervention, comparator, and outcome definitions.

In order to account for methodological differences between studies, we will perform analyses with two sets of studies: 1) the entire set of papers identified through this systematic review that meet the inclusion/exclusion criteria outlined above, and 2) the subset of studies identified through this systematic review that have more than 100 patients and meet a baseline level of methodological rigor. We have defined methodological rigor as studies that are either randomized controlled trials or observational studies that employ statistical approaches to adjust for differences in level of acute illness between patients in the intervention and control groups. We believe that statistical adjustment for acute level of illness is critical since patients that get follow-up blood cultures are often much different (e.g., much sicker) than patients that do not get follow-up blood cultures. Multiple methods for statistical adjustment are permissible, including propensity score-based approaches, exact matching techniques, or multivariable logistic regression. Statistical adjustment should be made, at minimum, for level of acute illness. Acute illness can be determined through validated scoring approaches (e.g., Pitt bacteremia score, APACHE-II score, SOFA score) or through individual clinical characteristics (e.g., ICU admission, need for vasopressor support, need for ventilatory support, etc.).

F. Grading the evidence of the key question

The strength of evidence for the key question will be assessed using the approach described in AHRQ's "Methods Guide." In brief, this approach requires assessment of four domains: risk of bias, consistency, directness, and precision. Additional domains are to be used when appropriate: coherence, impact of plausible residual confounders, strength of association (magnitude of effect), and publication bias. These domains will be

© 2022 Thaden JT et al. *JAMA Network Open.*

considered qualitatively, and a summary rating will be assigned after discussion by two providers as high, moderate, or low strength of evidence. In some cases, high, moderate, or low ratings will be impossible or imprudent to make. In these situations, a grade of insufficient will be assigned.

eAppendix 2. Search Strategies. Below are the search strategies and study results for searching the MEDLINE, Embase, Web of Science, and Cochrane Central Register of Controlled Trials databases. The initial searches were conducted on 2/11/2022. Repeat searches were performed on 3/11/2022 to identify any studies published while the systematic review was being conducted. In addition to the searches below, we manually searched the new journal *Antimicrobial Stewardship and Healthcare Epidemiology* as it was not indexed in the above databases at the time of this review (n=69) and IDWeek 2021 abstracts (*Open Forum Infectious Diseases*, Volume 8, Issue Supplement_1, 2021; n=1656). Librarian searcher: Sarah Cantrell, MLIS; Duke University Medical Center Library & Archives, Duke University School of Medicine. Peer-review conducted by: Brandi Tuttle, MSLS; Duke University Medical Center Library & Archives, Duke University School of Medicine

Database: MEDLINE (via Ovid)
Original search date: 2/11/2022
Note: Ovid MEDLINE(R) ALL 1946 to February 10, 2022

Search Set	Search Strategy	Results
#1 *Gram-negative bacteria terms*	exp Gram-Negative Bacteria/ OR exp Gram-Negative Bacterial Infections/ OR ("gram negative" OR gram-negative OR GNB OR GNBs OR enterobacteriaceae OR enterobacter OR "bacillary dysentery" OR escherichia OR "E.coli" OR "E. coli." OR "E coli" OR "granuloma inguinale" OR klebsiella OR rhinoscleroma OR proteus OR salmonella OR "typhoid fever" OR "paratyphoid fever" OR serratia OR yersinia OR citrobacer OR morganella OR providencia OR stenotrophomonas OR moraxellaceae OR acinetobacter OR mycoplasmatales OR mycoplasma OR ureaplasma OR "contagious pleuropneumonia" OR neisseriaceae OR gonorrhea OR meningococcal OR meningitis OR meningococcemia OR "neisseria meningitidis" OR "opthalmia neonatorum" OR pasteurellaceae OR actinobacillus OR actinobacillosis OR haemophilus OR chancroid OR "haemophilus meningitis" OR pasteurella OR "hemorrhagic septicemia" OR "hemorrhagic septicaemia" OR "haemorrhagic septicaemia" OR "pneumonic pasteurellosis" OR pseudomonas).ti,ab,kw,kf.	1227804
#2 *Bacteremia terms*	Bacteremia/ OR (bacteremia OR bacteraemia OR bacteremic OR bacteraemic OR "bloodstream infection" OR "blood stream infection" OR "bloodstream infections" OR "blood stream infections" OR "blood infection" OR "blood infections" OR pyemia OR pyemias OR pyohemia OR pyohemias OR pyaemia).ti,ab,kw,kf.	54,611
#3 *blood culture terms*	exp Blood culture/ OR (culture or cultures or cultured).ti,ab,kw,kf.	1103054
#4 *Follow-up terms*	(follow-up OR "follow up" OR "following up" OR "follow ups" OR follow-ups OR followup OR followups OR "followed up" OR "followed-up" OR repeat OR repeated OR repeating OR repeats OR repetitive OR repetitively OR "next day" OR "following day" OR serial OR serially OR surveillance OR surveil OR surveils OR surveilled OR surveilling OR FUBC OR FUBCs).ti,ab,kw,kf.	2040370
#5	3 and 4	51805
#6 *Add'l blood culture follow-up terms*	((culture OR cultures OR cultured) ADJ3 (frequent or frequently or daily)).ti,ab,kw,kf.	1804
#7	5 or 6	53415
#8	1 and 2 and 7	1034
#9 *Remove animal studies*	8 not (exp animals/ not exp humans/)	997
#10*Remove certain study designs*	9 not (case reports OR editorial OR congress).pt.	831

Search Update:
Database: MEDLINE (via Ovid)
Search date: 3/11/2022
Note: Ovid MEDLINE(R) ALL 1946 to March 10, 2022

Search Set	Search Strategy	Results
#1 *Gram-negative bacteria terms*	exp Gram-Negative Bacteria/ OR exp Gram-Negative Bacterial Infections/ OR ("gram negative" OR gram-negative OR GNB OR GNBs OR enterobacteriaceae OR enterobacter OR "bacillary dysentery" OR escherichia OR "E.coli" OR "E. coli." OR "E coli" OR "granuloma inguinale" OR klebsiella OR rhinoscleroma OR proteus OR salmonella OR "typhoid fever" OR "paratyphoid fever" OR serratia OR yersinia OR citrobacer OR morganella OR providencia OR stenotrophomonas OR moraxellaceae OR acinetobacter OR mycoplasmatales OR mycoplasma OR ureaplasma OR "contagious pleuropneumonia" OR neisseriaceae OR gonorrhea OR meningococcal OR meningitis OR meningococcemia OR "neisseria meningitidis" OR "opthalmia neonatorum" OR pasteurellaceae OR actinobacillus OR actinobacillosis OR haemophilus OR chancroid OR "haemophilus meningitis" OR pasteurella OR "hemorrhagic septicemia" OR "hemorrhagic septicaemia" OR "haemorrhagic septicaemia" OR "pneumonic pasteurellosis" OR pseudomonas).ti,ab,kw,kf.	1231002
#2 *Bacteremia terms*	Bacteremia/ OR (bacteremia OR bacteraemia OR bacteremic OR bacteraemic OR "bloodstream infection" OR "blood stream infection" OR "bloodstream infections" OR "blood stream infections" OR "blood infection" OR "blood infections" OR pyemia OR pyemias OR pyohemia OR pyohemias OR pyaemia).ti,ab,kw,kf.	54814
#3 *blood culture terms*	exp Blood culture/ OR (culture or cultures or cultured).ti,ab,kw,kf.	1105862
#4 *Follow-up terms*	(follow-up OR "follow up" OR "following up" OR "follow ups" OR follow-ups OR followup OR followups OR "followed up" OR "followed-up" OR repeat OR repeated OR repeating OR repeats OR repetitive OR repetitively OR "next day" OR "following day" OR serial OR serially OR surveillance OR surveil OR surveils OR surveilled OR surveilling OR FUBC OR FUBCs).ti,ab,kw,kf.	2049338
#5	3 and 4	52039
#6 *Add'l blood culture follow-up terms*	((culture OR cultures OR cultured) ADJ3 (frequent or frequently or daily)).ti,ab,kw,kf.	1812
#7	5 or 6	53658
#8	1 and 2 and 7	1042
#9 *Remove animal studies*	8 not (exp animals/ not exp humans/)	1005
#10 *Remove certain study designs*	9 not (case reports OR editorial OR congress).pt.	838
#11	Limit 10 to da=20220211-20220311	10

© 2022 Thaden JT et al. *JAMA Network Open.*

Database: Embase (via Elsevier)
Search date: 2/11/2022
Note: search from the Results page

Search Set	Search Strategy	Results
#1 *Gram-negative bacteria terms*	'Gram negative bacterium'/exp OR 'Gram negative infection'/exp OR ("gram negative" OR gram-negative OR GNB OR GNBs OR enterobacteriaceae OR enterobacter OR "bacillary dysentery" OR escherichia OR "E.coli" OR "E. coli." OR "E coli" OR "granuloma inguinale" OR klebsiella OR rhinoscleroma OR proteus OR salmonella OR "typhoid fever" OR "paratyphoid fever" OR serratia OR yersinia OR citrobacer OR morganella OR providencia OR stenotrophomonas OR moraxellaceae OR acinetobacter OR mycoplasmatales OR mycoplasma OR ureaplasma OR "contagious pleuropneumonia" OR neisseriaceae OR gonorrhea OR meningococcal OR meningitis OR meningococcemia OR "neisseria meningitidis" OR "opthalmia neonatorum" OR pasteurellaceae OR actinobacillus OR actinobacillosis OR haemophilus OR chancroid OR "haemophilus meningitis" OR pasteurella OR "hemorrhagic septicemia" OR "hemorrhagic septicaemia" OR "haemorrhagic septicaemia" OR "pneumonic pasteurellosis" OR pseudomonas):ti,ab,kw	1267935
#2 *Bacteremia terms*	'bacteremia'/exp OR (bacteremia OR bacteraemia OR bacteremic OR bacteraemic OR 'bloodstream infection' OR 'blood stream infection' OR 'bloodstream infections' OR 'blood stream infections' OR 'blood infection' OR 'blood infections' OR pyemia OR pyemias OR pyohemia OR pyohemias OR pyaemia):ti,ab,kw	87601
#3 *blood culture terms*	'blood culture'/exp OR (culture OR cultures OR cultured):ti,ab,kw	1452437
#4 *Follow-up terms*	(follow-up OR 'follow up' OR 'following up' OR 'follow ups' OR follow-ups OR followup OR followups OR 'followed up' OR 'followed-up' OR repeat OR repeated OR repeating OR repeats OR repetitive OR repetitively OR 'next day' OR 'following day' OR serial OR serially OR surveillance OR surveil OR surveils OR surveilled OR surveilling OR FUBC OR FUBCs):ti,ab,kw	3090369
#5 *combining*	#3 AND #4	84227
#6 *Add'l blood culture follow-up terms*	((culture OR cultures OR cultured) NEAR/3 (frequent or frequently or daily)):ti,ab,kw	2623
#7	#5 OR #6	86530
#8 *combining*	#1 AND #2 AND #7	2203
#9 *Remove animal studies*	#8 AND [humans]/lim	1976
#10 *Remove certain study designs*	#9 NOT ('case report'/exp OR 'editorial'/exp OR [editorial]/lim OR [conference abstract]/lim OR 'conference abstract'/exp OR 'conference abstract'/it)	978

© 2022 Thaden JT et al. *JAMA Network Open.*

Search update:
Database: Embase (via Elsevier)
Search date: 3/11/2022
Note: Search from the results page

Search Set	Search Strategy	Results
#1 *Gram-negative bacteria terms*	'Gram negative bacterium'/exp OR 'Gram negative infection'/exp OR ("gram negative" OR gram-negative OR GNB OR GNBs OR enterobacteriaceae OR enterobacter OR "bacillary dysentery" OR escherichia OR "E.coli" OR "E. coli." OR "E coli" OR "granuloma inguinale" OR klebsiella OR rhinoscleroma OR proteus OR salmonella OR "typhoid fever" OR "paratyphoid fever" OR serratia OR yersinia OR citrobacer OR morganella OR providencia OR stenotrophomonas OR moraxellaceae OR acinetobacter OR mycoplasmatales OR mycoplasma OR ureaplasma OR "contagious pleuropneumonia" OR neisseriaceae OR gonorrhea OR meningococcal OR meningitis OR meningococcemia OR "neisseria meningitidis" OR "opthalmia neonatorum" OR pasteurellaceae OR actinobacillus OR actinobacillosis OR haemophilus OR chancroid OR "haemophilus meningitis" OR pasteurella OR "hemorrhagic septicemia" OR "hemorrhagic septicaemia" OR "haemorrhagic septicaemia" OR "pneumonic pasteurellosis" OR pseudomonas):ti,ab,kw	1272600
#2 *Bacteremia terms*	'bacteremia'/exp OR (bacteremia OR bacteraemia OR bacteremic OR bacteraemic OR 'bloodstream infection' OR 'blood stream infection' OR 'bloodstream infections' OR 'blood stream infections' OR 'blood infection' OR 'blood infections' OR pyemia OR pyemias OR pyohemia OR pyohemias OR pyaemia):ti,ab,kw	88113
#3 *blood culture terms*	'blood culture'/exp OR (culture OR cultures OR cultured):ti,ab,kw	1456773
#4 *Follow-up terms*	(follow-up OR 'follow up' OR 'following up' OR 'follow ups' OR follow-ups OR followup OR followups OR 'followed up' OR 'followed-up' OR repeat OR repeated OR repeating OR repeats OR repetitive OR repetitively OR 'next day' OR 'following day' OR serial OR serially OR surveillance OR surveil OR surveils OR surveilled OR surveilling OR FUBC OR FUBCs):ti,ab,kw	3105757
#5 *combining*	#3 AND #4	84633
#6 *Add'l blood culture follow-up terms*	((culture OR cultures OR cultured) NEAR/3 (frequent or frequently or daily)):ti,ab,kw	2633
#7	#5 OR #6	86946
#8 *combining*	#1 AND #2 AND #7	2228
#9 *Remove animal studies*	#8 AND [humans]/lim	2000
#10 *Remove certain study designs*	#9 NOT ('case report'/exp OR 'editorial'/exp OR [editorial]/lim OR [conference abstract]/lim OR 'conference abstract'/exp OR 'conference abstract'/it)	985
#11	#10 AND [11-02-2022]/sd	15

© 2022 Thaden JT et al. *JAMA Network Open.*

Database: Web of Science – Science Citation Index Expanded (1900 – present) and Social Science Citation Index (1900-present) (via Clarivate)
Search date: 2/11/2022
Note: select indices from homepage, under Editions; search from the Advanced page

Search Set	Search Strategy	Results
#1 *Gram-negative bacteria terms*	TS=("gram negative" OR gram-negative OR GNB OR GNBs OR enterobacteriaceae OR enterobacter OR "bacillary dysentery" OR escherichia OR "E.coli" OR "E. coli." OR "E coli" OR "granuloma inguinale" OR klebsiella OR rhinoscleroma OR proteus OR salmonella OR "typhoid fever" OR "paratyphoid fever" OR serratia OR yersinia OR citrobacer OR morganella OR providencia OR stenotrophomonas OR moraxellaceae OR acinetobacter OR mycoplasmatales OR mycoplasma OR ureaplasma OR "contagious pleuropneumonia" OR neisseriaceae OR gonorrhea OR meningococcal OR meningitis OR meningococcemia OR "neisseria meningitidis" OR "opthalmia neonatorum" OR pasteurellaceae OR actinobacillus OR actinobacillosis OR haemophilus OR chancroid OR "haemophilus meningitis" OR pasteurella OR "hemorrhagic septicemia" OR "hemorrhagic septicaemia" OR "haemorrhagic septicaemia" OR "pneumonic pasteurellosis" OR pseudomonas)	933674
#2 *Bacteremia terms*	TS=(bacteremia OR bacteraemia OR bacteremic OR bacteraemic OR "bloodstream infection" OR "blood stream infection" OR "bloodstream infections" OR "blood stream infections" OR "blood infection" OR "blood infections" OR pyemia OR pyemias OR pyohemia OR pyohemias OR pyaemia)	57205
#3 *blood culture terms*	TS=(culture or cultures or cultured)	1358832
#4 *Follow-up terms*	TS=(follow-up OR "follow up" OR "following up" OR "follow ups" OR follow-ups OR followup OR followups OR "followed up" OR "followed-up" OR repeat OR repeated OR repeating OR repeats OR repetitive OR repetitively OR "next day" OR "following day" OR serial OR serially OR surveillance OR surveil OR surveils OR surveilled OR surveilling OR FUBC OR FUBCs)	2123891
#5	#3 AND #4	52215
#6 *Add'l blood culture follow-up terms*	TS=((culture OR cultures OR cultured) NEAR/3 (frequent or frequently or daily))	2244
#7	#5 OR #6	54221
#8	#1 AND #2 AND #7	1091
#9	#8 NOT Document Types: Book Chapters or Editorial Materials	1087

© 2022 Thaden JT et al. *JAMA Network Open.*

Search update:

Database: Web of Science – Science Citation Index Expanded (1900 – present) and Social Science Citation Index (1900-present) (via Clarivate)

Search date: 3/11/2022

Note: select indices from homepage, under Editions; search from the Advanced page

Search Set	Search Strategy	Results
#1 *Gram-negative bacteria terms*	TS=("gram negative" OR gram-negative OR GNB OR GNBs OR enterobacteriaceae OR enterobacter OR "bacillary dysentery" OR escherichia OR "E.coli" OR "E. coli." OR "E coli" OR "granuloma inguinale" OR klebsiella OR rhinoscleroma OR proteus OR salmonella OR "typhoid fever" OR "paratyphoid fever" OR serratia OR yersinia OR citrobacer OR morganella OR providencia OR stenotrophomonas OR moraxellaceae OR acinetobacter OR mycoplasmatales OR mycoplasma OR ureaplasma OR "contagious pleuropneumonia" OR neisseriaceae OR gonorrhea OR meningococcal OR meningitis OR meningococcemia OR "neisseria meningitidis" OR "opthalmia neonatorum" OR pasteurellaceae OR actinobacillus OR actinobacillosis OR haemophilus OR chancroid OR "haemophilus meningitis" OR pasteurella OR "hemorrhagic septicemia" OR "hemorrhagic septicaemia" OR "haemorrhagic septicaemia" OR "pneumonic pasteurellosis" OR pseudomonas)	936455
#2 *Bacteremia terms*	TS=(bacteremia OR bacteraemia OR bacteremic OR bacteraemic OR "bloodstream infection" OR "blood stream infection" OR "bloodstream infections" OR "blood stream infections" OR "blood infection" OR "blood infections" OR pyemia OR pyemias OR pyohemia OR pyohemias OR pyaemia)	57456
#3 *blood culture terms*	TS=(culture or cultures or cultured)	1362573
#4 *Follow-up terms*	TS=(follow-up OR "follow up" OR "following up" OR "follow ups" OR follow-ups OR followup OR followups OR "followed up" OR "followed-up" OR repeat OR repeated OR repeating OR repeats OR repetitive OR repetitively OR "next day" OR "following day" OR serial OR serially OR surveillance OR surveil OR surveils OR surveilled OR surveilling OR FUBC OR FUBCs)	2134139
#5	#3 AND #4	52414
#6 *Add'l blood culture follow-up terms*	TS=((culture OR cultures OR cultured) NEAR/3 (frequent or frequently or daily))	2251
#7	#5 OR #6	54426
#8	#1 AND #2 AND #7	1094
#9	#8 NOT Document Types: Book Chapters or Editorial Materials	1090
#10	Refine – Publication Year: 2022	10

© 2022 Thaden JT et al. *JAMA Network Open.*

Database: Cochrane Central Register of Controlled Trials (via Wiley)
Search date: 2/11/2021
Note: Go to Advanced Search > Search Manager

Search Set	Search Strategy	Results
#1 *Gram-negative bacteria terms*	[mh "Gram-Negative Bacteria"] OR [mh "Gram-Negative Bacterial Infections"] OR ("gram negative":ti,ab OR gram-negative:ti,ab OR GNB:ti,ab OR GNBs:ti,ab OR enterobacteriaceae:ti,ab OR enterobacter:ti,ab OR "bacillary dysentery":ti,ab OR escherichia:ti,ab OR E.coli:ti,ab OR "E. coli.":ti,ab OR "E coli":ti,ab OR "granuloma inguinale":ti,ab OR klebsiella:ti,ab OR rhinoscleroma:ti,ab OR proteus:ti,ab OR salmonella:ti,ab OR "typhoid fever":ti,ab OR "paratyphoid fever":ti,ab OR serratia:ti,ab OR yersinia:ti,ab OR citrobacer:ti,ab OR morganella:ti,ab OR providencia:ti,ab OR stenotrophomonas:ti,ab OR moraxellaceae:ti,ab OR acinetobacter:ti,ab OR mycoplasmatales:ti,ab OR mycoplasma:ti,ab OR ureaplasma:ti,ab OR "contagious pleuropneumonia":ti,ab OR neisseriaceae:ti,ab OR gonorrhea:ti,ab OR meningococcal:ti,ab OR meningitis:ti,ab OR meningococcemia:ti,ab OR "neisseria meningitidis":ti,ab OR "opthalmia neonatorum":ti,ab OR pasteurellaceae:ti,ab OR actinobacillus:ti,ab OR actinobacillosis:ti,ab OR haemophilus:ti,ab OR chancroid:ti,ab OR "haemophilus meningitis":ti,ab OR pasteurella:ti,ab OR "hemorrhagic septicemia":ti,ab OR "hemorrhagic septicaemia":ti,ab OR "haemorrhagic septicaemia":ti,ab OR "pneumonic pasteurellosis":ti,ab OR pseudomonas:ti,ab)	19373
#2 *Bacteremia terms*	[mh ^Bacteremia] OR (bacteremia:ti,ab OR bacteraemia:ti,ab OR bacteremic:ti,ab OR bacteraemic:ti,ab OR "bloodstream infection":ti,ab OR "blood stream infection":ti,ab OR "bloodstream infections":ti,ab OR "blood stream infections":ti,ab OR "blood infection":ti,ab OR "blood infections":ti,ab OR pyemia:ti,ab OR pyemias:ti,ab OR pyohemia:ti,ab OR pyohemias:ti,ab OR pyaemia:ti,ab)	3351
#3 *blood culture terms*	[mh "Blood culture"] OR (culture:ti,ab OR cultures:ti,ab OR cultured:ti,ab)	19359
#4 *Follow-up terms*	(follow-up:ti,ab OR "follow up":ti,ab OR "following up":ti,ab OR "follow ups":ti,ab OR follow-ups:ti,ab OR followup:ti,ab OR followups:ti,ab OR "followed up":ti,ab OR followed-up:ti,ab OR repeat:ti,ab OR repeated:ti,ab OR repeating:ti,ab OR repeats:ti,ab OR repetitive:ti,ab OR repetitively:ti,ab OR "next day":ti,ab OR "following day":ti,ab OR serial:ti,ab OR serially:ti,ab OR surveillance:ti,ab OR surveil:ti,ab OR surveils:ti,ab OR surveilled:ti,ab OR surveilling:ti,ab OR FUBC:ti,ab OR FUBCs:ti,ab)	312131
#5	#3 AND #4	4075
#6 *Add'l blood culture follow-up terms*	((culture:ti,ab OR cultures:ti,ab OR cultured:ti,ab) NEAR/3 (frequent:ti,ab OR frequently:ti,ab OR daily:ti,ab))	108
#7	#5 OR #6	4159
#8	#1 AND #2 AND #7	57
#9	From Results Page: Limit 8 to Trials	57

© 2022 Thaden JT et al. *JAMA Network Open.*

Search update:
Database: Cochrane Central Register of Controlled Trials (via Wiley)
Search date: 3/11/2022
Note: Go to Advanced Search > Search Manager

Search Set	Search Strategy	Results
#1 *Gram-negative bacteria terms*	[mh "Gram-Negative Bacteria"] OR [mh "Gram-Negative Bacterial Infections"] OR ("gram negative":ti,ab OR gram-negative:ti,ab OR GNB:ti,ab OR GNBs:ti,ab OR enterobacteriaceae:ti,ab OR enterobacter:ti,ab OR "bacillary dysentery":ti,ab OR escherichia:ti,ab OR E.coli:ti,ab OR "E. coli.":ti,ab OR "E coli":ti,ab OR "granuloma inguinale":ti,ab OR klebsiella:ti,ab OR rhinoscleroma:ti,ab OR proteus:ti,ab OR salmonella:ti,ab OR "typhoid fever":ti,ab OR "paratyphoid fever":ti,ab OR serratia:ti,ab OR yersinia:ti,ab OR citrobacer:ti,ab OR morganella:ti,ab OR providencia:ti,ab OR stenotrophomonas:ti,ab OR moraxellaceae:ti,ab OR acinetobacter:ti,ab OR mycoplasmatales:ti,ab OR mycoplasma:ti,ab OR ureaplasma:ti,ab OR "contagious pleuropneumonia":ti,ab OR neisseriaceae:ti,ab OR gonorrhea:ti,ab OR meningococcal:ti,ab OR meningitis:ti,ab OR meningococcemia:ti,ab OR "neisseria meningitidis":ti,ab OR "opthalmia neonatorum":ti,ab OR pasteurellaceae:ti,ab OR actinobacillus:ti,ab OR actinobacillosis:ti,ab OR haemophilus:ti,ab OR chancroid:ti,ab OR "haemophilus meningitis":ti,ab OR pasteurella:ti,ab OR "hemorrhagic septicemia":ti,ab OR "hemorrhagic septicaemia":ti,ab OR "haemorrhagic septicaemia":ti,ab OR "pneumonic pasteurellosis":ti,ab OR pseudomonas:ti,ab)	19804
#2 *Bacteremia terms*	[mh ^Bacteremia] OR (bacteremia:ti,ab OR bacteraemia:ti,ab OR bacteremic:ti,ab OR bacteraemic:ti,ab OR "bloodstream infection":ti,ab OR "blood stream infection":ti,ab OR "bloodstream infections":ti,ab OR "blood stream infections":ti,ab OR "blood infection":ti,ab OR "blood infections":ti,ab OR pyemia:ti,ab OR pyemias:ti,ab OR pyohemia:ti,ab OR pyohemias:ti,ab OR pyaemia:ti,ab)	3370
#3 *blood culture terms*	[mh "Blood culture"] OR (culture:ti,ab OR cultures:ti,ab OR cultured:ti,ab)	19447
#4 *Follow-up terms*	(follow-up:ti,ab OR "follow up":ti,ab OR "following up":ti,ab OR "follow ups":ti,ab OR follow-ups:ti,ab OR followup:ti,ab OR followups:ti,ab OR "followed up":ti,ab OR followed-up:ti,ab OR repeat:ti,ab OR repeated:ti,ab OR repeating:ti,ab OR repeats:ti,ab OR repetitive:ti,ab OR repetitively:ti,ab OR "next day":ti,ab OR "following day":ti,ab OR serial:ti,ab OR serially:ti,ab OR surveillance:ti,ab OR surveil:ti,ab OR surveils:ti,ab OR surveilled:ti,ab OR surveilling:ti,ab OR FUBC:ti,ab OR FUBCs:ti,ab)	314425
#5	#3 AND #4	4085
#6 *Add'l blood culture follow-up terms*	((culture:ti,ab OR cultures:ti,ab OR cultured:ti,ab) NEAR/3 (frequent:ti,ab OR frequently:ti,ab OR daily:ti,ab))	108
#7	#5 OR #6	4169
#8	#1 AND #2 AND #7	59
#9	From Results Page: Limit 8 to Trials	59
#10	Limit to Year 2022	2

© 2022 Thaden JT et al. *JAMA Network Open.*

eAppendix 3. Criteria for inclusion and exclusion of studies in this systematic review.

Inclusion criteria

1. Study design: Randomized controlled trials or observational studies with N>10. The sample size requirement is designed to exclude case reports. This size requirement does permit smaller pilot studies that are typically underpowered and have more methodological problems than larger studies.
2. For observational studies, matching or statistical adjustment for differences in (at minimum) level of acute illness between patients in the intervention (e.g., FUBCs obtained) and control groups (e.g., FUBCs not obtained) must be performed. Only observational studies meeting this criteria were included in the primary analyses for KQ1 and KQ2. However, observational studies not meeting this criteria were included in exploratory analyses of KQ1 and KQ2.
3. Age: Adults aged 18 years or older with gram-negative bloodstream infection
4. Setting: Inpatients
5. Studies must stratify patients by whether follow-up blood cultures obtained (KQ1) or by whether follow-up blood cultures were positive or negative (KQ2)
6. Study must report effects of follow-up blood cultures on at least one of the following relevant outcomes:
    - In-hospital mortality
    - Mortality up to 30 days (e.g., 28-day mortality, 30-day mortality)
7. Article is a full publication, research letter, or meeting abstract published in a peer-reviewed journal.
8. Article is an English language publication

Exclusion criteria

1. Outpatient setting
2. Polymicrobial bloodstream infection

© 2022 Thaden JT et al. *JAMA Network Open.*

eAppendix 4. Studies excluded at full text review.

1. Alsfeld LC, Rockey DC. Utility of Routine Blood Cultures for Inpatient Hematology/Oncology Patients Receiving Antimicrobials. American Journal of the Medical Sciences 2019; 358: 175-81. Reason for exclusion: Wrong outcome.

2. Canzoneri CN, Akhavan BJ, Andrade PEA et al. Prospective Trials Are Required to Alter Practice for Follow-up Blood Cultures for Gram-Negative Bacilli Bacteremia Reply. Clinical Infectious Diseases 2018; 67: 316-. Reason for exclusion: Commentary without data.

3. Canzoneri CN, Akhavan BJ, Tosur Z et al. Follow-up Blood Cultures in Gram-Negative Bacteremia: Are They Needed? Clinical Infectious Diseases 2017; 65: 1776-9. Reason for exclusion: Data not stratified by gram-negative bacteria.

4. Canzoneri CN, Akhavan BJ, Tosur Z et al. Follow-up Blood Cultures: A 2.0 Diagnostic Tool in Patients With Gram-Negative Bacteremia and Septic Thrombophlebitis Reply. Clinical Infectious Diseases 2018; 66: 1155-6. Reason for exclusion: Commentary without data.

5. Ceccarelli G, Giuliano S, Falcone M et al. Follow-up Blood Cultures: A 2.0 Diagnostic Tool in Patients With Gram-Negative Bacteremia and Septic Thrombophlebitis. Clinical Infectious Diseases 2018; 66: 1154-5. Reason for exclusion: Case report.

6. Chan JD, Bryson-Cahn C, Kassamali-Escobar Z et al. The Changing Landscape of Uncomplicated Gram-Negative Bacteremia: A Narrative Review to Guide Inpatient Management. Journal of Hospital Medicine (Online) 2020; 15: 746-53. Reason for exclusion: Reason for exclusion: Review article.

7. Cogliati Dezza F, Curtolo A, Volpicelli L et al. Are Follow-Up Blood Cultures Useful in the Antimicrobial Management of Gram Negative Bacteremia? A Reappraisal of Their Role Based on Current Knowledge. Antibiotics 2020; 9: 11. Reason for exclusion: Review article.

8. Deal EN, Micek ST, Reichley RM et al. Effects of an alternative cefepime dosing strategy in pulmonary and bloodstream infections caused by Enterobacter spp, Citrobacter freundii, and Pseudomonas aeruginosa: a single-center, open-label, prospective, observational study. Clinical Therapeutics 2009; 31: 299-310. Reason for exclusion: Wrong intervention.

9. Fabre V, Sharara SL, Salinas AB et al. Does this patient need blood cultures? A scoping review of indications for blood cultures in adult nonneutropenic inpatients. Clinical Infectious Diseases 2020; 71: 1339-47. Reason for exclusion: Review article.

10. Funada H, Machi T, Matsuda T. [Pseudomonas aeruginosa bacteremia associated with hematologic disorders [II]. Blood culture isolates and surveillance cultures]. Kansenshogaku Zasshi - Journal of the Japanese Association for Infectious Diseases 1989; 63: 874-9. Reason for exclusion: Article not in English.

11. Giannella M, Malosso P, Scudeller L et al. Quality of care indicators in the MAnageMent of BlOOdstream infections caused by Enterobacteriaceae (MAMBOO-E study): state of the art and research agenda. International journal of antimicrobial agents 2021; 57: 106320. Reason for exclusion: Review article.

12. Gubbels S, Nielsen J, Voldstedlund M et al. Utilization of blood cultures in Danish hospitals: a population-based descriptive analysis. Clinical Microbiology and Infection 2015; 21. Reason for exclusion: Wrong patient population.

13. Harris PNA, Peri AM, Pelecanos AM et al. Risk factors for relapse or persistence of bacteraemia caused by Enterobacter spp.: a case-control study. Antimicrobial Resistance & Infection Control 2017; 6: 14. Reason for exclusion: Wrong outcomes.

14. Jafri F, Knoll BM. Low diagnostic yield of repeat blood cultures in adult haematologic malignancy patients with persistent neutropenic fever. Journal of internal medicine 2021; 289: 584-7. Reason for exclusion: Wrong intervention.

15. Jones RB, Paruchuri A, Shah SS. Prospective Trials Are Required to Alter Practice for Follow-up Blood Cultures for Gram-Negative Bacilli Bacteremia. Clinical Infectious Diseases 2018; 67: 315-6. Reason for exclusion: Commentary without data.

16. Kimura SI, Gomyo A, Hayakawa J et al. Clinical significance of repeat blood cultures during febrile neutropenia in adult acute myeloid leukaemia patients undergoing intensive chemotherapy. Infectious Diseases 2017; 49: 748-57. Reason for exclusion: Wrong patient population.

17. Lee CC, Yang CY, Hsieh CC et al. Timing of follow-up blood cultures for community-onset bacteremia. Scientific reports 2019; 9. Reason for exclusion: Data not stratified by gram-negative bacteria.

18. Limmathurotsakul D, Wuthiekanun V, Wongsuvan G et al. Short report: Repeat blood culture positive for B. pseudomallei indicates an increased risk of death from melioidosis. American Journal of Tropical Medicine and Hygiene 2011; 84: 858-61. Reason for exclusion: Wrong patient population.

19. McKinnell JA, Connolly LE, Pushkin R et al. Improved outcomes with plazomicin (PLZ) compared with colistin (CST) in patients with bloodstream infections (BSI) caused by carbapenem-resistant enterobacteriaceae (CRE): results from the care study. Open forum infectious diseases 2017; 4: S531-. Reason for exclusion: Wrong intervention.

20. Pastewski AA, Caruso P, Parris AR et al. Parenteral polymyxin B use in patients with multidrug-resistant gram-negative bacteremia and urinary tract infections: a retrospective case series. Annals of Pharmacotherapy 2008; 42: 1177-87. Reason for exclusion: Wrong outcomes.

21. Paterson DL, Kinoshita M, Baba T et al. Outcomes with Cefiderocol Treatment in Patients with Bacteraemia Enrolled into Prospective Phase 2 and Phase 3 Randomised Clinical Studies. Infectious Diseases and Therapy. Reason for exclusion: Wrong study design.

22. Shi HJ, Kang CI, Cho SY et al. Follow-up blood cultures add little value in the management of bacteremic urinary tract infections. European Journal of Clinical Microbiology and Infectious Diseases 2019; 38: 695-702. Reason for exclusion: Data not stratified by gram-negative bacteria.

23. Shi HJ, Lee JS, Cho YK et al. Predictors of Mortality in Patients with Carbapenem-Resistant Gram-Negative Bacilli or Vancomycin-Resistant Enterococci Bacteremia. Infection & Drug Resistance 2020; 13: 3535-42. Reason for exclusion: Data not stratified by gram-negative bacteria.

24. Spaziante M, Giuliano S, Ceccarelli G et al. Gram-negative septic thrombosis in critically ill patients: A retrospective case-control study. International Journal of Infectious Diseases 2020; 94: 110-5. Reason for exclusion: Wrong intervention.

25. Sutton JD, Sayood S, Spivak ES. Top questions in uncomplicated, non-staphylococcus aureus Bacteremia. Open forum infectious diseases 2018; 5. Reason for exclusion: Review article.

26. Trachtenberg BH, Cordero-Reyes AM, Aldeiri M et al. Persistent blood stream infection in patients supported with a continuous-flow left ventricular assist device is associated with an increased risk of cerebrovascular accidents. Journal of Cardiac Failure 2015; 21: 119-25. Reason for exclusion: Data not stratified by gram-negative bacteria.

27. Wang YL, Scipione MR, Dubrovskaya Y. Monotherapy with Fluoroquinolone or Trimethoprim-Sulfamethoxazole for Treatment of Stenotrophomonas maltophilia Infections. Antimicrobial agents and chemotherapy 2014; 58: 176-82. Reason for exclusion: Wrong outcomes.

28. Wiggers JB, Daneman N. The culture of follow-up blood cultures. Clinical Microbiology & Infection 2020; 26: 811-3. Reason for exclusion: Review article.

29. Zmora N, Shrestha S, Neuberger A et al. Open label comparative trial of mono versus dual antibiotic therapy for Typhoid Fever in adults. PLoS Neglected Tropical Diseases [electronic resource] 2018; 12: e0006380. Reason for exclusion: Wrong comparator.

© 2022 Thaden JT et al. *JAMA Network Open.*

eAppendix 5. Newcastle-Ottawa Quality Assessment Scale for assessing risk of bias in cohort (A) and case-control (B) studies. The detailed criteria used to assess these study types, as well as the thresholds for converting the Newcastle-Ottawa scale to AHRQ standards (good, fair, and poor), are shown below.

A. Cohort studies

Selection

1. Representativeness of the exposed cohort
   a. Truly representative of the average person with gram-negative bloodstream infection in the community (*)
   b. Somewhat representative of the average person with gram-negative bloodstream infection in the community (*)
   c. Selected group of patients
   d. No description of the derivation of the cohort

2. Selection of the non-exposed cohort
   a. Drawn from the same community as the exposed cohort (*)
   b. Drawn from a different source
   c. No description of the derivation of the non-exposed cohort

3. Ascertainment of exposure
   a. Secure record (e.g. medical records) (*)
   b. Structured interview (*)
   c. Written self report
   d. No description

4. Demonstration that outcome of interest was not present at start of study
   a. Yes (*)
   b. No

Comparability of cohorts on basis of design or analysis

1. Study controls for level of acute illness.
   a. Yes (*)
   b. No

2. Study controls for any additional factor.
   a. Yes (*)
   b. No

Outcome

1. Assessment of outcome
   a. Independent blind assessment (*)
   b. Record linkage (*)
   c. Self report
   d. No description

2. Was follow-up long enough for outcomes to occur
   a. Yes (In-hospital mortality or mortality ≤30 days) (*)
   b. No

3. Adequacy of follow up of cohorts
   a. Complete follow up (all subjects accounted for) (*)

© 2022 Thaden JT et al. *JAMA Network Open.*

b. Subjects lost to follow up unlikely to introduce bias (≤10% lost to follow-up, or description provided of those lost) (*)
c. Follow up rate < 90% and no description of those lost
d. No statement

Thresholds used to convert the Newcastle-Ottawa scale to AHRQ standards (good, fair, and poor):

Good quality/low risk of bias: 3 or 4 stars in selection domain AND 1 or 2 stars in comparability domain AND 2 or 3 stars in outcome/exposure domain

Fair quality/medium risk of bias: 2 stars in selection domain AND 1 or 2 stars in comparability domain AND 2 or 3 stars in outcome/exposure domain.

Poor quality/high risk of bias: 0 or 1 star in selection domain OR 0 stars in comparability domain OR 0 or 1 stars in outcome/exposure domain

B. Case-control studies

Selection

1. Is the case definition adequate?
   a. Yes, with independent validation (*)
   b. Yes, with record linkage or based on self-reports
   c. No description

2. Representativeness of the cases
   a. Consecutive or obviously representative series of cases (*)
   b. Potential for selection biases or not stated

3. Selection of Controls
   a. Community controls (*)
   b. Hospital controls
   c. No description

4. Definition of Controls
   a. No history of disease (endpoint) (*)
   b. No description of source

Comparability of cases and controls on the basis of the design or analysis

1. Study controls for level of acute illness.
   a. Yes (*)
   b. No

2. Study controls for any additional factor.
   a. Yes (*)
   b. No

Exposure

1. Ascertainment of exposure
   a. Secure record (e.g. medical records) (*)
   b. Structured interview where blind to case/control status (*)
   c. Interview not blinded to case/control status
   d. Written self-report or medical record only
   e. No description

© 2022 Thaden JT et al. *JAMA Network Open.*

2. Same method of ascertainment for cases and controls
    a. Yes (*)
    b. No

3. Non-Response rate
    a. Same rate for both groups (*)
    b. Non respondents described
    c. Rate different and no designation

Thresholds used to convert the Newcastle-Ottawa scale to AHRQ standards (good, fair, and poor):

Good quality/low risk of bias: 3 or 4 stars in selection domain AND 1 or 2 stars in comparability domain AND 2 or 3 stars in outcome/exposure domain

Fair quality/medium risk of bias: 2 stars in selection domain AND 1 or 2 stars in comparability domain AND 2 or 3 stars in outcome/exposure domain.

Poor quality/high risk of bias: 0 or 1 star in selection domain OR 0 stars in comparability domain OR 0 or 1 stars in outcome/exposure domain

© 2022 Thaden JT et al. *JAMA Network Open.*

eTable 1. Detailed description of studies that address KQ1 and KQ2.

Detailed characteristics of studies that addressed KQ1.

Study	Design	Patient Population	Micro	Country	# Sites	Number With FUBCs	Number Without FUBCs	Patient Characteristics With FUBCs	Patient Characteristics Without FUBCs	Outcome
Amipara 2021[3]	Cohort	Adult inpatients with community-acquired BSI	GN bacilli	USA	2	168[a]	438[a]	Age (Median [IQR]) 65 (52-75)<br>Female 56%<br>Pitt bacteremia score: Median (IQR) 2 (1-3)	Age: Median (IQR) 68 (56-79)<br>Female 58%<br>Pitt bacteremia score: Median (IQR) 1 (1-3)	28-day mortality
Chan 2021[5]	Cohort	All adult inpatients	*E. coli* and *Klebsiella*	USA	1	299	36	Age (Median [IQR]) 60 (48-71)<br>Female 42%	Age (Median [IQR]) 64 (53-72)<br>Female 67%	30-day mortality
Clemmons 2021[6]	Cohort	Non-critically ill adults in oncology units	All GN	USA	3	35	17	Age: Mean (SD) 59 (14)<br>Female 49%	Age: Mean (SD) 71 (17)<br>Female 53%	In-hospital mortality
Giannella 2020[2]	Cohort	All adult inpatients	All GN	Italy	1	278	1298	Age: Mean (SD) 62 (16)<br>Female 49%<br>SOFA: Mean (SD) 3 (1-5)<br>Septic shock 14%	Age: Mean (SD) 70 (16)<br>Female 46%<br>SOFA: Mean (SD) 3 (1-5)<br>Septic shock 8%	30-day mortality
Green 2021[7]	Cohort	All adult inpatients	*P. aeruginosa*	USA	1	127	32	Age: Mean (SD) 57 (17)<br>Female 40%<br>Pitt bacteremia score: Median (IQR) 2 (0-4)	Age: Mean (SD) 59 (15)<br>Female 44%<br>Pitt bacteremia score: Median (IQR) 3 (0.5-5.5)	30-day mortality
Groft 2021[8]	Cohort	Immuno-compromised adults	All GN	USA	1	117	22	ICU 41%<br>Neutropenic 32%	ICU 41%<br>Neutropenic 50%	In-hospital mortality
Jung 2020[4]	Cohort	All adult inpatients	All GN	South Korea	1	1276	205	Age: Mean (SD) 69 (14)<br>Female 51%	Age: Mean (SD) 69 (14)<br>Female 43%	In-hospital mortality
Maskarinec 2020[1]	Cohort	All adult inpatients	All GN	USA	2	1164	538	Age: Mean (SD) 60 (16)<br>Female 43%<br>APACHE-II: Mean (SD) 7 (6)	Age: Mean (SD) 62 (16)<br>Female 46%<br>APACHE-II: Mean (SD) 7 (5)	In-hospital
Mitaka 2021[9]	Cohort	All adult inpatients	All GN	USA	4	276	105	Age: Median (IQR) 69 (59-82)<br>Female 55%	Age: Median (IQR) 73 (64-83)<br>Female 51%	In-hospital mortality
Robinson 2021[10]	Cohort	All adult inpatients	All GN	USA	1	338	176	Age: Median (IQR) 63 (52-72)	Age: Median (IQR) 67 (57-76)	In-hospital mortality
Wiggers 2016[11]	Cohort	All adult inpatients	All GN	Canada	1	247	654	Not reported	Not reported	30-day mortality

© 2022 Thaden JT et al. *JAMA Network Open.*

Detailed characteristics of studies that addressed KQ1 (continued).

Study	Mortality With FUBCs n (%)	Mortality Without FUBCs n (%)	Timing of FUBC from index culture	Matching/ adjustment	Covariates Matched/ Adjusted (selected)	Adjusted Estimate of Mortality with FUBCs (95% CI)	Method(s) to Address Survival Bias	Study Funding	Conflict Of Interest	Risk of bias
Amipara 2021[3]	12 (7.1)	57 (13.0)	24-96h	IPTW	Age, Charlson comorbidity score, Pitt bacteremia score, antibiotic therapy, FUBC	HR 0.47 (0.23-0.87)	Excluded patients that died or were discharged <72h from index culture	None	None	Low
Chan 2021[5]	27 (9.0)	5 (13.9)	24h-7d	None	None	None	Excluded patients that died <48h or discharged <24h after index culture	None	None	High
Clemmons 2021[6]	3 (8.6)	1 (5.9)	4-72h	None	None	None	None reported	None	None	High
Giannella 2020[2]	38 (13.7)	126 (9.7)	24h-7d	Logistic regression and matching on SOFA score (separately performed)	Charlson comorbidity score, SOFA score, septic shock, urinary tract source, source control, antibiotic therapy, FUBC	Logistic regression: HR 0.48 (0.27-0.83)<br><br>Matching: HR 0.45 (0.22-0.92)	FUBCs treated as time-dependent covariate; patients with and without FUBCs matched by SOFA score, and those without FUBCs survived at least as long as it took for matched patient to get FUBCs; Excluded patients that died <72h from index culture	None	None	Low
Green 2021[7]	24 (18.9)	16 (50.0)	24h-7d	Cox regression	Age, Elixhauser comorbidity index, Pitt bacteremia score, immunosuppression	HR 0.43 (0.17-1.08)	FUBCs treated as time-dependent variable in Cox model	IDSA/SHEA	None	Low
Groft 2021[8]	7 (6.0)	1 (4.5)	24h-7d	None	None	None	None reported	None	None	High
Jung 2020[4]	87 (7)	18 (9)	2-7d	None	None	None	Excluded patients that died <2 days from index culture	None	None	High

© 2022 Thaden JT et al. *JAMA Network Open.*

Study	Mortality With FUBCs n (%)	Mortality Without FUBCs n (%)	Timing of FUBC from index culture	Matching/ adjustment	Covariates Matched/ Adjusted (selected)	Adjusted Estimate of Mortality with FUBCs (95% CI)	Method(s) to Address Survival Bias	Study Funding	Conflict Of Interest	Risk of bias
Maskarinec 2020[1]	176 (15)	108 (20)	24h-7d	IPTW	Age, APACHE-II acute physiology score, diabetes mellitus, transplant, hemodialysis, source of BSI, antibiotic therapy	HR 0.63 (0.51-0.72)	FUBCs treated as time-dependent variable in Cox model; Excluded patients that died <24h from index culture (sensitivity analysis with patients that died <48h from index culture performed and no change in findings)	None	None	Low
Mitaka 2021[9]	4 (4.6)[c]	10 (11.5)[c]	≥24h	Propensity score matching	Not reported[b]	OR 0.36 (0.12-1.14)	Excluded patients that received hospice care or died within 3 days of index culture	None	None	Low
Robinson 2021[10]	338 (6.5)	176 (5.7)	24h-7d	None	None	None	Excluded patients that died or were discharged <24h from index culture	None	None	Low
Wiggers 2016[11]	35 (14.2)[d]	96 (14.7)[d]	2-7d	None	None	None	Excluded patients that died <2 days from index culture	Canadian Institutes of Health Research	None	High

[a]Censored patients (n=51 in FUBCs group; n=109 in No FUBCs group) were removed to generate these numbers.
[b]Specific covariates not described in the conference abstract, though two intervention/control arms matched with respect to acute illness parameters (e.g., vasopressor support, ventilatory support, ICU level of care)
[c]Mortality figures only provided for propensity score-matched cohort
[d]Mortality data from this study was further stratified by gram-negative bacteria in other reports [12,13]
Abbreviations: CI, confidence interval; FUBCs, follow-up blood cultures; GN, gram-negative; HR, hazard ratio; IPTW, inverse probability of treatment weighting; IQR, interquartile range; OR, odds ratio; SD, standard deviation

© 2022 Thaden JT et al. *JAMA Network Open.*

Detailed characteristics of studies that addressed KQ2.

Study	Design	Patient Population	Micro	Country	# Sites	Number With Positive FUBCs	Number With Negative FUBCs	Patient Characteristics With Positive FUBCs	Patient Characteristics With Negative FUBCs	Outcome
Amipara 2021[3]	Cohort	Adult inpatients with community-acquired BSI	GN bacilli	USA	2	13[a]	155[a]	Not reported	Not reported	28-day mortality
Chan 2021[5]	Cohort	All adult inpatients	*E. coli* and *Klebsiella*	USA	1	37	262	Age: Median (IQR) 54 (44-67)<br>Female 46%	Age: Median (IQR) 60 (48-71)<br>Female 41%	30-day mortality
Groft 2021[8]	Cohort	Immuno-compromised adults	All GN	USA	1	3	114	Not reported	Not reported	In-hospital mortality
Kang 2013[14]	Case control	All adult inpatients	*K. pneumoniae*	South Korea	2	62	186	Age: Mean (SD) 64 (15)<br>Female 27%<br>APACHE-II score: Mean (SD) 19 (9)<br>Pitt bacteremia score: Mean (SD) 3 (3)	Age: Mean (SD) 64 (14)<br>Female 27%<br>APACHE-II score: Mean (SD) 17 (8)<br>Pitt bacteremia score: Mean (SD) 2 (2)	In-hospital mortality
Kim 2022[15]	Cohort	All adult inpatients	All GN	South Korea	1	89	556	Age: Median (IQR) 60 (49-67)<br>Female 41%<br>Septic shock 47%	Age: Median (IQR) 64 (55-73)<br>Female 27%<br>Septic shock 30%	30-day mortality
Maskarinec 2020[1]	Cohort	All adult inpatients	All GN	USA	2	228	885	Age: Mean (SD) 59 (15)<br>Female 46%<br>APACHE-II score: Mean (SD) 8 (5)	Age: Mean (SD) 60 (16)<br>Female 45%<br>APACHE-II score: Mean (SD) 7 (5)	In-hospital
Mitaka 2020[16]	Cohort	All adult inpatients	All GN	USA	4	28	278	Age: Median (IQR) 66 (57-77)<br>Female 43%<br>Septic shock 29%	Age: Median (IQR) 70 (59-83)<br>Female 46%<br>Septic shock 23%	In-hospital
Spaziante 2020[17]	Cohort	Critically ill adult inpatients	All GN	Italy	1	23	26	Age: Mean (SD) 55 (41-66)<br>Female 30%<br>SOFA score: Median (IQR) 7 (5-10)	Age: Mean (SD) 61 (44-73)<br>Female 19%<br>SOFA score: Median (IQR) 9 (7-10)	30-day mortality
Wiggers 2016[11]	Cohort	All adult inpatients	All GN	Canada	1	27	220	Not reported	Not reported	30-day mortality

© 2022 Thaden JT et al. *JAMA Network Open.*

Detailed characteristics of studies that addressed KQ2 (continued).

Study	Mortality With Positive FUBCs n (%)	Mortality With Negative FUBCs n (%)	Timing of FUBC from index culture	Matching/ adjustment	Covariates Matched/ Adjusted (selected)	Adjusted Estimate of Mortality with Positive FUBCs (95% CI)	Study Funding	Conflict Of Interest	Risk of bias
Amipara 2021[3]	2 (15.4)	10 (6.5)	24-96h	None	None	None	None	None	Low
Chan 2021[5]	6 (16.2)	21 (8.0)	24h-7d	None	None	None	None	None	High
Groft 2021[8]	1 (33.3)	6 (5.3)	24h-7d	None	None	None	None	None	High
Kang 2013[14]	19 (30.6)	34 (18.3)	Not reported	None	None	None	None	None	Low
Kim 2022[15]	31 (34.8)	55 (9.9)	48-72h	Logistic regression	Age, need for HD dependence, positive FUBC, stem cell transplant, need for vasopressor, antibiotic therapy	OR 2.01 (1.07-3.76)	Asan Institute for Life Sciences	None	Low
Maskarinec 2020[1]	49 (21.5)	110 (12.4)	24h-7d	IPTW	Age, APACHE-II acute physiology score, diabetes mellitus, transplant, hemodialysis, source of BSI, antibiotic therapy	HR 1.56 (1.13-2.16)	None	None	Low
Mitaka 2020[16]	3 (10.7)	25 (9.0)	≥24h	None	None	None	None	None	High
Spaziante 2020[17]	8 (40.0)	12 (46.1)	<48h after antibiotics started	None	None	None	None	None	High
Wiggers 2016[11]	7 (25.9)[b]	28 (12.7)[b]	2-7d	None	None	None	Canadian Institutes of Health Research	None	High

[a]Censored patients with positive FUBCs (n=2 with positive FUBCs and n=49 with negative FUBCs) were removed to generate these numbers. The number of censored patients were provided by authors.
[b]Mortality data from this study was further stratified by gram-negative bacteria in other reports[12,13]
Abbreviations: BSI, bloodstream infection; CI, confidence interval; GN, gram-negative; HR, hazard ratio; IPTW, inverse probability of treatment weighting; IQR, interquartile range; OR, odds ratio; SD, standard deviation

© 2022 Thaden JT et al. *JAMA Network Open.*

eAppendix 6. Risk of bias (quality) assessment of included cohort (A) and case-control (B) studies using the Newcastle-Ottawa Quality Assessment Scale. The Newcastle-Ottawa Quality Assessment Scale determines a study's risk of bias through nine questions (detailed in eAppendix 6). For each study, the grades for the nine questions are shown below. Grades that receive a star are highlighted in green, while those that do not are highlighted in red. Based on the grades from each question in the Newcastle-Ottawa Scale, an overall risk of bias (high, medium, low) can be assigned (detailed in eAppendix 6).

A. Cohort studies

**Study**	**Selection:** Representativeness of the exposed cohort	**Selection:** Selection of non-exposed cohort	**Selection:** Ascertainment of exposure	**Selection:** Outcome not present at start of study	**Comparability:** Controls for level of acute illness	**Comparability:** Controls for any additional factor	**Outcome:** Assessment of outcome	**Outcome:** Was follow-up long enough	**Outcome:** Adequacy of follow-up	**Risk of bias**
**Amipara 2021**	B	A	A	A	A	A	B	A	C	Low
**Chan 2021**	C	A	A	A	B	B	B	A	A	High
**Clemmons 2021**	C	A	A	A	B	B	B	A	A	High
**Giannella 2020**	B	A	A	A	A	A	B	A	A	Low
**Green 2021**	C	A	A	A	A	A	B	A	B	Low
**Groft 2021**	C	A	A	A	B	B	B	A	A	High
**Jung 2020**	B	A	A	A	B	B	B	A	A	High
**Kim 2022**	B	A	A	A	A	A	B	A	A	Low
**Maskarinec 2020**	B	A	A	A	A	A	B	A	A	Low
**Mitaka 2020**	B	A	A	A	B	B	B	A	A	High
**Mitaka 2021**	B	A	A	A	A	A	B	A	A	Low
**Robinson 2021**	B	A	A	A	B	B	B	A	A	High
**Spaziante 2020**	C	A	A	A	B	B	B	A	A	High
**Wiggers 2016**	B	A	A	A	B	B	B	A	A	High

© 2022 Thaden JT et al. *JAMA Network Open.*

B. Case control studies

Study	**Selection:** Is case definition adequate?	**Selection:** Representativeness of the cases	**Selection:** Selection of controls	**Selection:** Definition of controls	**Comparability:** Controls for level of acute illness	**Comparability:** Controls for any additional factor	**Exposure:** Ascertainment of exposure	**Exposure:** Same method for ascertainment of cases and controls	**Exposure:** Non-response rate	**Risk of bias**
**Kang 2013**	**A**	**A**	**A**	**A**	**B**	**A**	**A**	**A**	**A**	**Low**

© 2022 Thaden JT et al. *JAMA Network Open.*

eFigure 1. Funnel plot for Key Question (KQ) 1. A funnel plot with studied that address KQ1 did not reveal evidence of publication bias (Egger's test p-value = 0.59). There were less than 10 studies that addressed KQ2, so a funnel plot was not generated.

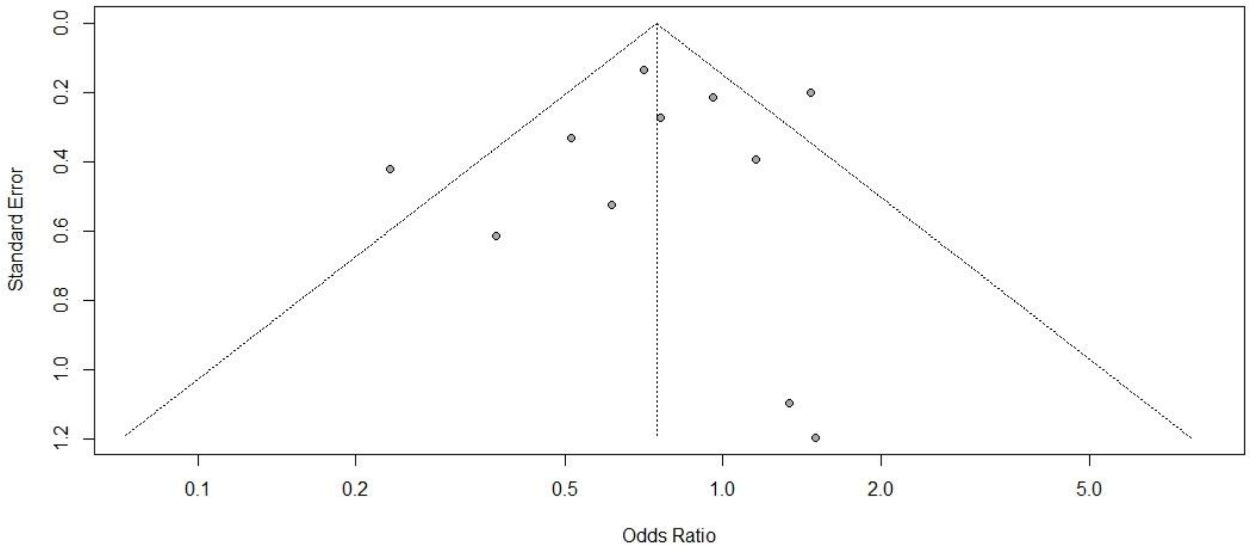

© 2022 Thaden JT et al. *JAMA Network Open.*

eFigure 2. Robustness of KQ1 primary analysis. (A) Studies included in the KQ1 primary analysis (n=5) were stratified by whether full study results were published in a peer-reviewed manuscript (n=4) or whether data obtained from a conference abstract (n=1). A meta-analysis of the four studies with full results in a peer-reviewed manuscript is shown. The mortality hazard ratio is listed, with hazard ratio less than 1 indicating decreased mortality with obtaining follow-up blood cultures [FUBCs]. (B) Giannella et al. performed two adjusted KQ1 analyses. In the first, patients with and without FUBCs were matched 1:1 based on sequential Organ Failure Assessment [SOFA] scores, and patients without FUBCs survived at least to the time when FUBCs were obtained in the matched patient. The data from this analysis was incorporated into the meta-analysis shown in Figure 2. In the second analysis, the influence of FUBCs on mortality was addressed with a multivariable Cox regression model. The data from this second analysis was incorporated into the meta-analysis in this figure. There were no major differences in the results from the two meta-analyses. (C) An influence analysis showed that the overall result (i.e., decreased mortality with FUBCs) did not change with omission of any single study. All five studies in the KQ1 primary analysis had good quality (i.e., low risk of bias) and so no further stratification by study quality was necessary. **Abbreviations:** CI, confidence interval; HR, hazard ratio.

A

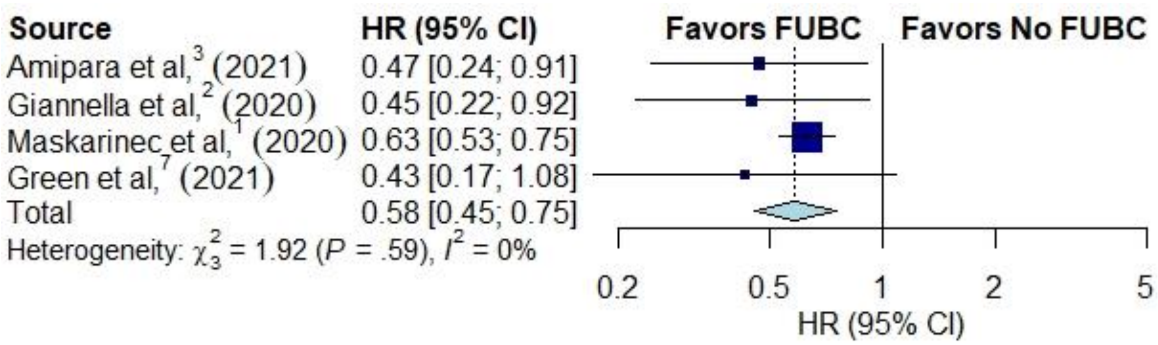

B

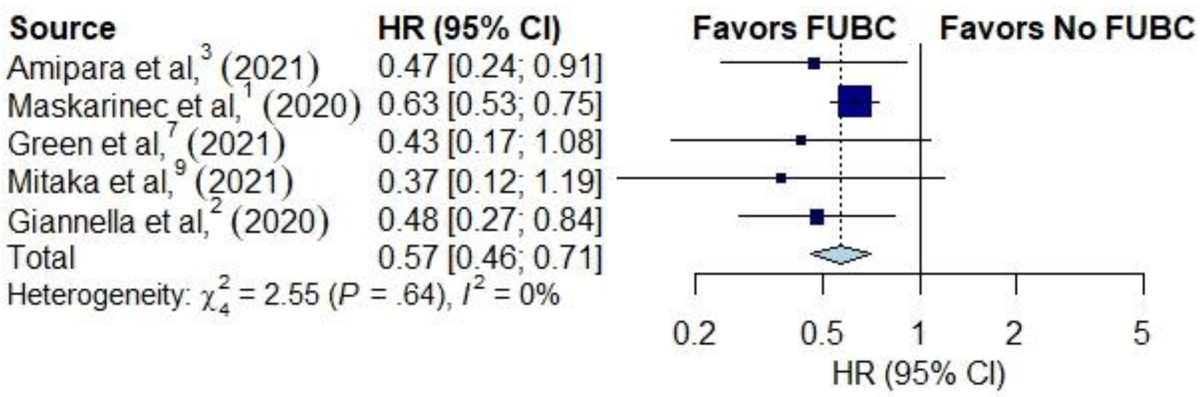

C

	HR	95% CI	p-value	tau^2	tau	I^2
Omitting Amipara 2021	0.5778	0.4277; 0.7806	0.0102	0.0125	0.1118	0.0%
Omitting Giannella 2020	0.6062	0.4985; 0.7386	0.0040	0.0000	0.0000	0.0%
Omitting Maskarinec 2020	0.4597	0.3796; 0.5568	0.0010	0.0000	0.0000	0.0%
Omitting Green 2021	0.6037	0.4926; 0.7398	0.0042	0.0001	0.0104	0.0%
Omitting Mitaka 2021	0.6034	0.4978; 0.7314	0.0036	0.0000	0.0000	0.0%
**Pooled estimate**	**0.5820**	**0.4757; 0.7121**	**0.0017**	**0.0060**	**0.0775**	**0.0%**

eFigure 3.. Exploratory analysis of KQ1 with unadjusted mortality data. (A) Meta-analysis of mortality odds ratios (OR) using unadjusted mortality data from all studies included in KQ1 (n=11). (B) Meta-analysis of mortality OR using unadjusted mortality data from all studies included in KQ1 except Giannella et al. (C) Influence analysis showed that omission of Giannella et al. substantially reduced the observed heterogeneity in the meta-analysis.
Abbreviation: CI, confidence interval; FUBC, follow-up blood culture.

A

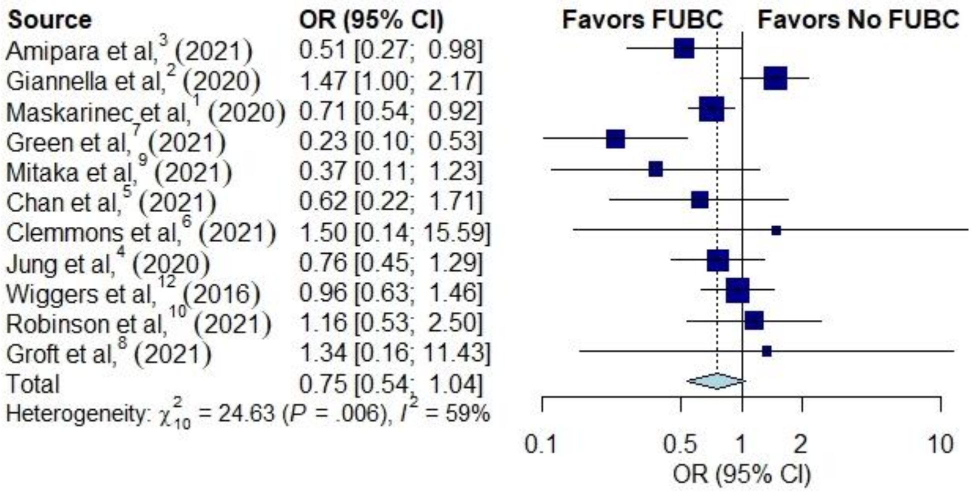

B

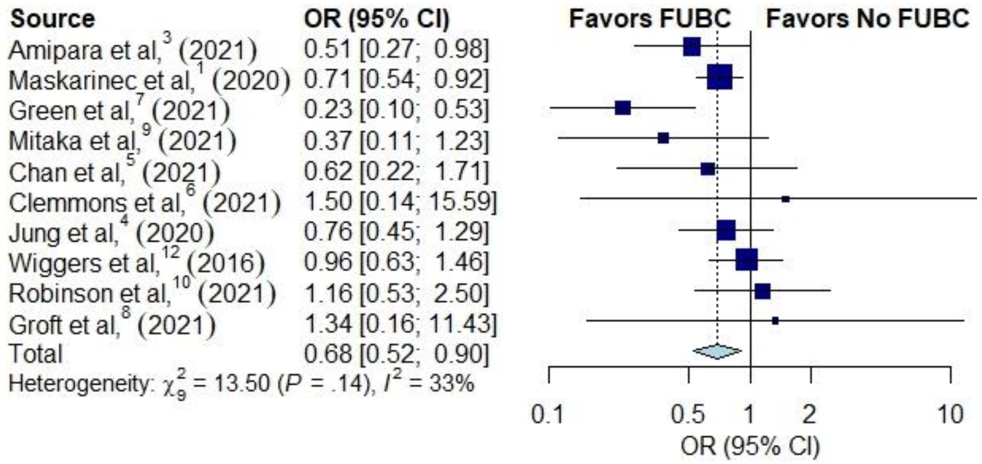

C

	OR	95% CI	p-value	tau^2	tau	I^2 (%)
Omitting Amipara 2021	0.7802	0.5459; 1.1151	0.1732	0.1713	0.4138	60.2
Omitting Giannella 2020	0.6845	0.5214; 0.8987	0.006	0.0587	0.2423	33.3
Omitting Maskarinec 2020	0.7472	0.5039; 1.1078	0.1470	0.2133	0.4619	61.1
Omitting Green 2021	0.8458	0.6449; 1.1093	0.2262	0.0713	0.2670	41.9
Omitting Mitaka 2021	0.7785	0.5551; 1.0919	0.1469	0.1581	0.3976	60.8
Omitting Chan 2021	0.7556	0.5289; 1.0794	0.1236	0.1816	0.4262	63.0
Omitting Clemmons 2021	0.7375	0.5257; 1.0345	0.0778	0.1675	0.4093	63.0
Omitting Jung 2020	0.7399	0.5048; 1.0845	0.1226	0.2052	0.4529	63.4
Omitting Wiggers 2016	0.7143	0.4883; 1.0448	0.0829	0.1971	0.4439	62.3
Omitting Robinson 2021	0.7132	0.4979; 1.0218	0.0654	0.1789	0.4230	62.1
Omitting Groft 2021	0.7375	0.5250; 1.0359	0.0790	0.1609	0.4107	63.1
**Pooled estimate**	**0.7482**	**0.5373; 1.0417**	**0.0858**	**0.1609**	**0.4012**	**59.4**

© 2022 Thaden JT et al. *JAMA Network Open.*

eFigure 4. Robustness of KQ2 exploratory analysis. (A) Meta-analysis of mortality odds ratios (OR) using unadjusted mortality data from all studies with cohort design (n=8). A single study with case-control design was excluded from this analysis. (B) Meta-analysis of mortality odds ratios using unadjusted mortality data from all studies that address KQ2 and have low risk of bias. (C) Influence analysis showed that general findings of the meta-analysis (i.e., increased odds of mortality with positive blood cultures relative to negative blood cultures) did not change with omission of any single study. **Abbreviation:** CI, confidence interval.

A Meta-analysis with cohort studies

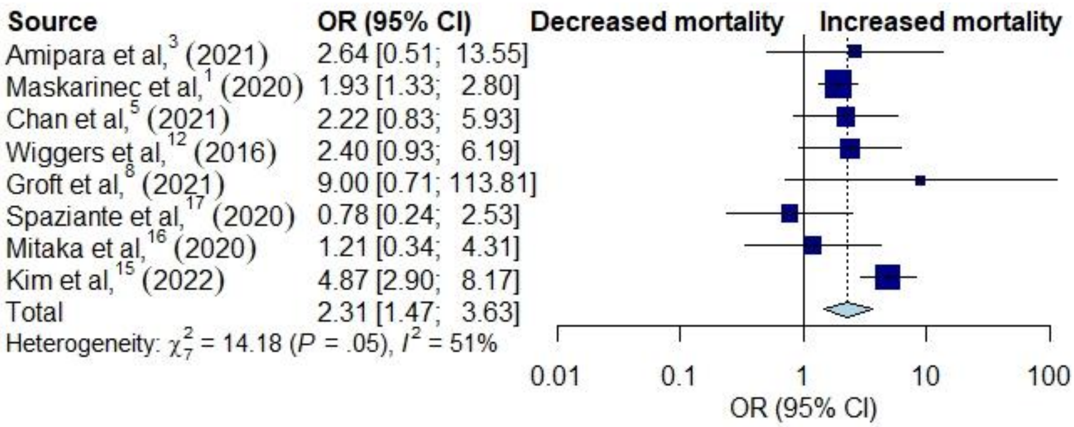

B Meta-analysis with studies rated as having low risk of bias

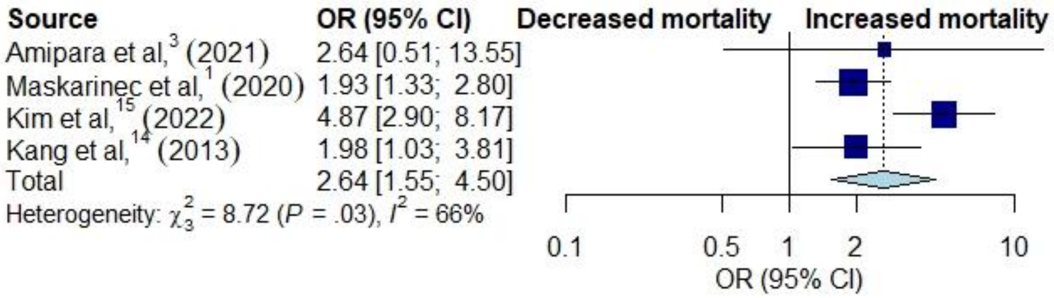

C Influence analysis

	OR	95% CI	p-value	tau^2	tau	I^2 (%)
Omitting Amipara 2021	2.2434	1.4931; 3.3708	0.0001	0.1491	0.3861	51.6
Omitting Maskarinec 2020	2.3422	1.4607; 3.7559	0.0004	0.1829	0.4277	45.0
Omitting Chan 2021	2.2581	1.4648; 3.4810	0.0002	0.1672	0.4089	51.6
Omitting Wiggers 2016	2.2361	1.4466; 3.4563	0.0003	0.1694	0.4116	51.6
Omitting Groft 2021	2.1975	1.4831; 3.2561	<0.0001	0.1376	0.3709	47.7
Omitting Spaziante 2020	2.4860	1.7092; 3.6158	<0.0001	0.1013	0.3183	36.3
Omitting Mitaka 2020	2.3769	1.5902; 3.5528	<0.0001	0.1356	0.3682	47.8
Omitting Kim 2022	1.9090	1.4542; 2.5061	<0.0001	0.0000	0.0010	0.0
Omitting Kang 2013	2.3056	1.4655; 3.6274	0.0003	0.1774	0.4212	50.6
**Pooled estimate**	**2.2678**	**1.5421; 3.3351**	**<0.0001**	**0.1332**	**0.3650**	**44.7**

© 2022 Thaden JT et al. *JAMA Network Open.*

eTable 2. Evidence profile for association of obtaining follow-up blood cultures with mortality in patients with gram-negative bloodstream infections.

**Patient population**	Gram-negative bloodstream infections
**Setting**	Hospital
**Intervention**	Follow-up blood cultures
**Comparison**	No follow-up blood cultures
**Outcome**	Mortality (In-hospital or up to 30 days)
**Studies (Participants)**	5 (4584)
**Risk of bias**	Low
**Consistency**	Consistent
**Precision**	Precise
**Directness**	Direct
**Other limitations**	No randomized controlled trials
**Overall strength of evidence**	Moderate
**Conclusions**	Follow-up blood cultures associated with decreased mortality<br>Hazard ratio 0.56 (95% Confidence interval 0.45 to 0.71)

© 2022 Thaden JT et al. *JAMA Network Open.*

eMethods. Approach for grading the overall strength of evidence.

We used the Evidence-based Practice Center (EPC) model from the U.S. Agency for Healthcare Research and Quality (AHRQ) to grade the overall strength of evidence.[18] The EPC approach evaluates the following domains: study limitations/risk of bias, consistency, directness, precision, and reporting bias. In brief, the EPC classification system applies an overall strength of evidence grade rating to an estimate effect from a body of evidence: high (we are very confident that the estimate of effect lies close to the true effect for this outcome), moderate (we are moderately confident that the estimate of effect lies close to the true effect for this outcome), low (we have limited confidence that the estimate of effect lies close to the true effect for this outcome), or insufficient (we have no evidence, we are unable to estimate an effect, or we have no confidence in the estimate of effect for this outcome). The initial strength of evidence grade was moderate given that the included observational studies in the primary analysis reduced bias from confounding through matching or statistical adjustment.[18] This baseline category could be rated down if the included studies demonstrated high risk of bias, imprecision, inconsistency, indirectness, or reporting bias.

© 2022 Thaden JT et al. *JAMA Network Open.*

eReferences

1. Maskarinec SA, Park LP, Ruffin F, et al. Positive follow-up blood cultures identify high mortality risk among patients with Gram-negative bacteraemia. *Clin Microbiol Infect*. Jul 2020;26(7):904-910. doi:10.1016/j.cmi.2020.01.025
2. Giannella M, Pascale R, Pancaldi L, et al. Follow-up blood cultures are associated with improved outcome of patients with gram-negative bloodstream infections: retrospective observational cohort study. *Clin Microbiol Infect*. Jul 2020;26(7):897-903. doi:10.1016/j.cmi.2020.01.023
3. Amipara R, Winders HR, Justo JA, Bookstaver PB, Kohn J, Al-Hasan MN. Impact of follow up blood cultures on outcomes of patients with community-onset gram-negative bloodstream infection. *EClinicalMedicine*. Apr 2021;34:100811. doi:10.1016/j.eclinm.2021.100811
4. Jung J, Song KH, Jun KI, et al. Predictive scoring models for persistent gram-negative bacteremia that reduce the need for follow-up blood cultures: a retrospective observational cohort study. *BMC Infect Dis*. Sep 17 2020;20(1):680. doi:10.1186/s12879-020-05395-8
5. Chan JD, Ta A, Lynch JB, Bryson-Cahn C. Follow-up blood cultures in E. coli and Klebsiella spp. bacteremia-opportunities for diagnostic and antimicrobial stewardship. *Eur J Clin Microbiol Infect Dis*. May 2021;40(5):1107-1111. doi:10.1007/s10096-020-04141-x
6. Clemmons AB, Young HN, Bland CM, et al. Incidence and utility of follow-up blood cultures in cancer patients with gram-negative bacteremia. *Diagn Microbiol Infect Dis*. Oct 2021;101(2):115444. doi:10.1016/j.diagmicrobio.2021.115444
7. Green AL, Liang Y, O'Hara LM, et al. Follow-up blood cultures in *Pseudomonas aeruginosa* bacteremia: A potential target for diagnostic stewardship. *Antimicrobial Stewardship & Healthcare Epidemiology*. 2021;1(1)doi:10.1017/ash.2021.184
8. Groft L, Mease J, Bork J, Bernhardi CL, Kristie Johnson J, Claeys KC. 62. Follow-Up Blood Culture Practices for Gram-Negative Bloodstream Infections in Immunocompromised Hosts at a Large Academic Medical Center. *Open Forum Infectious Diseases*. 2021;8(Supplement_1):S42-S42. doi:10.1093/ofid/ofab466.062
9. Mitaka H, Kuno T, Fujitani S. 179. Follow-up Blood Cultures for Gram-negative Bacilli Bacteremia Were Associated with Prolonged Length of Hospital Stay and Duration of Antibiotic Treatment: A Propensity Score-matched Cohort Study. *Open Forum Infectious Diseases*. 2021;8(Supplement_1):S198-S198. doi:10.1093/ofid/ofab466.381
10. Robinson ED, Cox H, Mathers AJ. 89. Follow-Up Blood Cultures (FUBC) in the Management of Gram-Negative Bacilli (GNB) Bloodstream Infections (BSIs): Frequently Obtained and Rarely Helpful. *Open Forum Infectious Diseases*. 2021;8(Supplement_1):S160-S160. doi:10.1093/ofid/ofab466.291
11. Wiggers JB, Xiong W, Daneman N. Sending repeat cultures: is there a role in the management of bacteremic episodes? (SCRIBE study). *BMC Infect Dis*. Jun 13 2016;16:286. doi:10.1186/s12879-016-1622-z
12. Wiggers JB, Daneman N. The culture of follow-up blood cultures. *Clinical Microbiology & Infection*. 2020;26(7):811-813.
13. Giannella M, Malosso P, Scudeller L, et al. Quality of care indicators in the MAnageMent of BlOOdstream infections caused by Enterobacteriaceae (MAMBOO-E study): state of the art and research agenda. *International Journal of Antimicrobial Agents*. 2021;57(4):106320.
14. Kang CK, Kim ES, Song KH, et al. Can a routine follow-up blood culture be justified in *Klebsiella pneumoniae* bacteremia? A retrospective case-control study. *BMC Infect Dis*. Aug 2 2013;13:365. doi:10.1186/1471-2334-13-365
15. Kim H, Seo H, Chung H, et al. Bedside risk prediction for positive follow-up blood culture in Gram-negative bacilli bacteremia: for whom is follow-up blood culture useful? *Infection*. Jan 21 2022;doi:10.1007/s15010-021-01742-2
16. Mitaka H, Gomez T, Lee YI, Perlman DC. Risk Factors for Positive Follow-Up Blood Cultures in Gram-Negative Bacilli Bacteremia: Implications for Selecting Who Needs Follow-Up Blood Cultures. *Open Forum Infect Dis*. Apr 2020;7(4):ofaa110. doi:10.1093/ofid/ofaa110
17. Spaziante M, Oliva A, Ceccarelli G, Alessandri F, Pugliese F, Venditti M. Follow-up blood cultures in Gram-negative bacilli bacteremia: are they needed for critically ill patients? *Minerva Anestesiol*. May 2020;86(5):498-506. doi:10.23736/S0375-9393.20.14040-9
18. Berkman ND, Lohr KN, Ansari MT, et al. Grading the strength of a body of evidence when assessing health care interventions: an EPC update. *J Clin Epidemiol*. Nov 2015;68(11):1312-24. doi:10.1016/j.jclinepi.2014.11.023

© 2022 Thaden JT et al. *JAMA Network Open.*
